# Adjunctive posterior wall isolation for patients with persistent atrial fibrillation: A systematic review and meta-analysis

**DOI:** 10.1016/j.hroo.2024.12.008

**Published:** 2025-01-04

**Authors:** André Rivera, Antônio S. Menezes, Douglas Mesadri Gewehr, Bárbara Nascimento, Isabele Ayumi Miyawaki, Luís E. Rohde, Caique M.P. Ternes, Arash Aryana, André d’Avila

**Affiliations:** 1 Department of Medicine, Nove de Julho University, São Bernardo do Campo, Brazil; 2 Federal University of Goiás, Goiânia, Brazil; 3 Curitiba Heart Institute, Curitiba, Brazil; 4 Denton Cooley Institute of Research, Science, and Technology, Curitiba, Brazil; 5 Federal University of Rio Grande do Sul, Porto Alegre, Brazil; 6 Federal University of Paraná, Curitiba, Brazil; 7 Mercy General Hospital and Dignity Health Heart and Vascular Institute, Sacramento, California; 8 Harvard Thorndike Electrophysiology Institute, Beth Israel Deaconess Medical Center, Boston, Massachusetts

**Keywords:** Pulmonary vein isolation, Atrial fibrillation, Posterior wall isolation, Catheter ablation, Box isolation

## Abstract

**Background:**

Pulmonary vein isolation (PVI) is the cornerstone of atrial fibrillation (AF) ablation. Its effectiveness for persistent atrial fibrillation (PeAF) is limited, and the benefits of adjunctive posterior wall ablation are uncertain.

**Objective:**

The purpose of this study was to perform a systematic review and meta-analysis of PVI with/without adjunctive PWI in patients with PeAF.

**Methods:**

We systematically searched PubMed, Embase, Cochrane, and ClinicalTrials.gov databases for randomized controlled trials (RCTs) comparing PVI with/without PWI in patients with PeAF. Random-effects model was used for the meta-analysis. Atrial tachyarrhythmia (ATA) was a composite of AF, atrial flutter, or atrial tachycardia.

**Results:**

Our meta-analysis included eight RCTs with 1104 patients (546 PVI, 558 PVI plus PWI). Compared with PVI alone, adjunctive PWI significantly increased freedom from ATA recurrence (relative risk [RR] 1.13, 95% confidence interval [CI] 1.01–1.27, *P* = .036). A subanalysis of patients with long-standing PeAF showed a greater effect of PWI (RR 1.76, 95% CI 1.02–3.04, *P* = .04). A subgroup analysis of PWI techniques indicated no significant difference for ATA recurrence with box isolation alone (RR 1.13, 95% CI 0.97–1.33, *P* = .12), whereas a pooled analysis using only studies with direct posterior wall ablation favored the adjunctive PWI group (RR 1.39, 95% CI 1.11–1.74, *P* <.01). Adverse events did not significantly differ between groups.

**Conclusion:**

Our findings support that adjunctive PWI to PVI is an effective strategy compared with PVI alone for reducing ATA recurrence in patients with PeAF without compromising safety. Notably, patients with long-standing PeAF may benefit more from PWI.

## Graphical abstract

**Figure:**
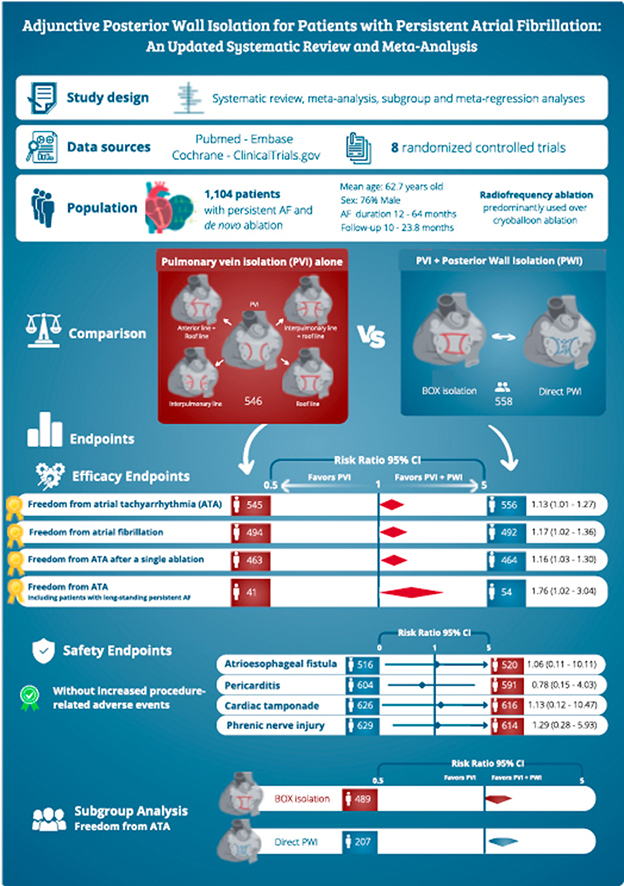

**Key Findings**

▪ Adjunctive posterior wall isolation (PWI) increased freedom from atrial tachyarrhythmias (ATAs) compared with pulmonary vein isolation alone (PVI) in patients with persistent atrial fibrillation (AF).
▪ There were no significant differences between groups with regard to in atrioesophageal fistula, pericarditis, cardiac tamponade, or phrenic nerve injury.
▪ There was a higher freedom from ATA associated with PWI among patients with long-standing persistent AF compared with PVI alone.

## Introduction

Atrial fibrillation (AF) ablation is an effective strategy for treating symptomatic AF, having been shown to be superior to medical therapy in reducing AF recurrence, symptomatic AF, and AF burden.^1^

Pulmonary vein isolation (PVI) has been the cornerstone of AF ablation,^2^ but its efficacy in patients with persistent atrial fibrillation (PeAF) is inferior to those with paroxysmal AF.^3^ Ablation strategies beyond PVI have emerged as frequently used adjunctive approaches, particularly in scenarios of reduced likelihood of success. However, the enthusiasm for extra-PVI ablation in patients with AF has been dampened because of the limited efficacy in several clinical trials.^4^, ^5^, ^6^ Accordingly, the 2020 European Society of Cardiology Guidelines for the diagnosis and management of AF has assigned a class 2B recommendation for additional ablation strategies beyond PVI.^1^

Previous meta-analyses have investigated the role of adjunctive posterior wall isolation (PWI) and shown lower recurrence of atrial tachyarrhythmias (ATAs) in patients with PeAF.^7^ This approach is frequently used during PeAF ablation as a nonpulmonary vein trigger because of the shared embryological origin of the pulmonary veins and the left atrial posterior wall.^8^ However, the recent CAPLA (Catheter Ablation for Persistent Atrial Fibrillation: A Multicenter Randomized Trial of Pulmonary Vein Isolation vs PVI With Posterior Left Atrial Wall Isolation) and PEF-HOT (Posterior Wall Isolation for Persistent Atrial Fibrillation High-Power Short Duration Trial) trials found that PVI had similar endpoints at 12 months, regardless of PWI.^9^^,^^10^ Furthermore, interest in this ablation strategy has increased with the emergence of pulsed field ablation, given its nonthermal nature and potential for improved safety.^11^

Given this uncertainty, we performed a systematic review and meta-analysis of randomized controlled trials (RCTs) to further investigate the efficacy and safety of PVI with adjunctive PWI compared with PVI alone in patients with PeAF.

## Methods

The systematic review and meta-analysis were performed and reported following the Cochrane Collaboration Handbook for Systematic Reviews of Interventions and the Preferred Reporting Items for Systematic Reviews and Meta-Analysis (PRISMA) Statement guidelines (Supplemental Methods 1).^12^^,^^13^ The prospective meta-analysis protocol was uploaded to the International Prospective Register of Systematic Reviews (PROSPERO; CRD42023397998).

### Data source and search strategy

We systematically searched PubMed (MEDLINE), Scopus, Cochrane, and ClinicalTrials.gov from inception to February 2023. The search terms used included “pulmonary vein isolation”, “posterior wall isolation”, and “atrial fibrillation”. The complete search strategy is provided in Supplemental Methods 2. Two authors (AR, BN) independently screened titles and abstracts and evaluated the articles in full for eligibility based on prespecified criteria. Discrepancies were resolved in a panel discussion with the senior author. Moreover, we used backward snowballing (ie, review of references) to identify relevant texts from articles identified in the original search.

### Eligibility criteria

We considered studies eligible for inclusion if they (1) were RCTs; (2) compared PVI alone vs PVI plus adjuvant PWI; (3) enrolled patients with PeAF (defined as a sustainable episode lasting ≥7 days) or long-standing PeAF (defined as continuous AF >12 months’ duration) undergoing *de novo* ablation; and (4) presented data regarding prespecified efficacy and safety endpoints. Our exclusion criteria were studies enrolling only patients with paroxysmal AF, studies not confirming PVI with a circular mapping catheter, conference abstracts, or reviews. Randomized trials allowing operators to perform additional ablations (eg, anterior line, roof line, interpulmonary line ablation) were not excluded.

### Data extraction

Two authors (AR, BN) independently extracted the data for each study using a standardized study form to determine: authors, clinical trial registration number, enrollment period (Supplemental Methods 3), study publication year, main exclusion criteria (Supplemental Methods 4), sample size, follow-up period, baseline patient characteristics, antiarrhythmic drug (AAD) use at baseline, PVI and PWI techniques used, endpoint definitions, the methods used to confirm electrical isolation during ablation (Supplemental Methods 5), and the posterior wall reconnection rates at repeated catheter ablation. Discrepancies were resolved in a panel discussion with the senior author.

### Endpoints

Our primary efficacy endpoint was freedom from ATA, defined as AF, atrial flutter, or atrial tachycardia. Prespecified secondary efficacy endpoints included (1) freedom from AF; (2) freedom from atrial flutter/tachycardia; (3) freedom from ATA after a single ablation procedure; (4) freedom from ATA without AAD; (5) freedom from ATA after a single ablation procedure without AAD; (6) need for cardioversion; and (7) need for repeat ablation. We performed a subanalysis of freedom from ATA restricted to patients with long-standing PeAF. Prespecified procedure-related endpoints consisted of (1) total procedural time; (2) ablation time; and (3) fluoroscopy time. Prespecified safety endpoints included (1) pericarditis; (2) cardiac tamponade; (3) phrenic nerve injury; and (4) atrioesophageal fistula. Other secondary endpoints were (1) short-term (<12 months) and long-term (≥12 months) AAD use and (2) change in left atrial diameter (mm). Supplemental Methods 6 comprehensively describes the endpoint definitions and methods used for each study.

### Subgroup and meta-regression analysis

We conducted prespecified subgroup and meta-regression analyses for the primary endpoint. Studies were grouped based on (1) PWI technique (direct posterior wall ablation vs box isolation); (2) the technique used for catheter ablation (radiofrequency catheter ablation vs cryoballoon ablation); (3) overall risk of bias; and (4) study location. In addition, we added a sensitivity analysis omitting studies that performed an additional mitral linear ablation in the adjunctive PWI group. We performed a meta-regression to assess for interactions between the outcomes and study-patient characteristics, including (1) duration of AF; (2) left atrial diameter; (3) mean age of patients; and (4) left ventricular ejection fraction. Box isolation was defined as the creation of a box in the posterior wall, doing roof and bottom lines, whereas direct posterior wall ablation was defined as the ablation of the posterior wall itself.

### Quality assessment

Two independent authors assessed the risk of bias in the included RCTs using the Cochrane tool for assessing the risk of bias in randomized trials (RoB 2).^12^ Any disagreements were resolved by consensus. We explored the potential for publication bias through funnel plots and the Egger test for the primary endpoint.

### Statistical analysis

We summarized binary endpoints using the Mantel-Haenszel random-effects model (restricted maximum likelihood estimator for t^2^) with risk ratio (RR) and 95% confidence interval (CI) as a measure of effect size. Furthermore, we used weighted mean differences (MD) to pool continuous endpoints. To assess absolute risk difference, we performed an analysis with risk difference (RD) and 95% CI. We assessed heterogeneity with the Cochrane Q statistic and Higgins and Thompson I^2^ statistic, with *P* ≤.10 indicating statistical significance. We determined the consistency of the studies based on I^2^ values of 0%, ≤25%, ≤50%, and >50%, indicating no observed low, moderate, and substantial heterogeneity, respectively. All tests were 2-tailed, and *P* <.05 was considered significant. If necessary, the mean and SD were estimated.^14^ We used R Version 4.2.2 and the extension packages "meta" for all calculations and graphics.^15^ An in-depth description of the statistical analyses is available in Supplemental Methods 7.

## Results

### Study selection and characteristics

**Figure 1:**
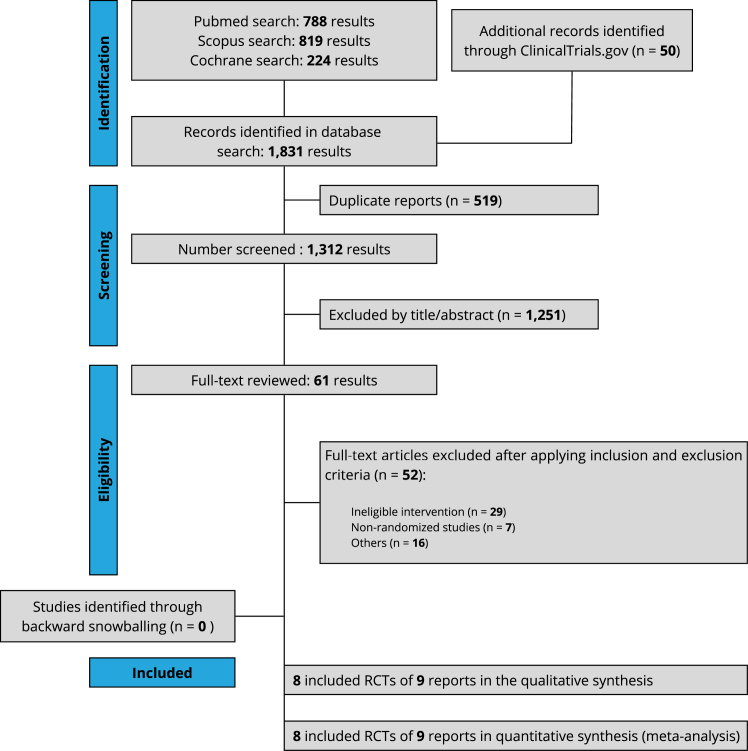
PRISMA flow diagram of study screening and selection. RCT= randomized controlled trial.

Our systematic search yielded 1831 potential articles (Figure 1). After removing duplicates, 61 articles were retrieved and reviewed in full for possible inclusion. Of these, 8 RCTs met all inclusion criteria and were included in the primary analysis.^9^^,^^10^^,^^16^, ^17^, ^18^, ^19^, ^20^, ^21^ We included 1104 patients, with 546 patients (49.5%) assigned to PVI alone and 558 patients (50.5%) assigned to PVI with PWI. Table 1 summarizes the main characteristics of the included studies. Supplemental Tables 1 and 2 summarize the clinical baseline characteristics of the patients and the AADs used before randomization.

**Table 1:** Baseline characteristics of included studies

First author, year (study acronym or registry)	Country (RCT identifier)	No. of patients	Ablation procedure	PVI group ablation technique	Adjunctive PWI group ablation technique	Mean duration of AF, mo	Follow-up,∗ mo
Kistler, 2023 (CAPLA)	AUS, CA, and UK (ACTRN12616001436460)	338	Radiofrequency ablation	Circumferential PVI without interpulmonary isthmus line	BOX isolation ± debulking (red points on LA posterior wall)	26.29	12
Wong, 2023 (PEF-HOT)	USA (—)	67	High-power short duration	Circumferential PVI with interpulmonary isthmus line	BOX isolation ± debulking (red points on LA posterior wall)	12	12.4
Ahn, 2022	Korea (KCT0004149)	100	Cryoballoon ablation	Circumferential PVI without interpulmonary isthmus line	Direct PWI	56.15	15
Aryana, 2021	Japan and USA (NCT03057548)	110	Cryoballoon ablation with adjunct radiofrequency ablation	Circumferential PVI without interpulmonary isthmus line + CTI	Direct PWI	NA	21
Pak, 2021 (PEACEFUL)	Korea (NCT02176616)	114	Radiofrequency ablation	Circumferential PVI with interpulmonary isthmus line + CTI	BOX isolation	31.74	23.8
Lee, 2019 (POBI-AF)	Korea (NCT02721121)	207	Radiofrequency ablation	Circumferential PVI with interpulmonary isthmus line + CTI	BOX isolation	38.5	16.2
Yu, 2017	Korea (NCT02176616)	113	Radiofrequency ablation	Circumferential PVI without interpulmonary isthmus line + CTI	BOX isolation	42.8	18.6
Kim, 2015	Korea (—)	120	Radiofrequency ablation	Circumferential PVI without interpulmonary isthmus line + LA roof line + anterior wall LA + CTI	BOX isolation	NA	12
Tamborero, 2009	Spain (—)	48	Radiofrequency ablation	Circumferential PVI without interpulmonary isthmus line + LA roof line ablation + mitral isthmus ablation	BOX isolation	63.9†	10

AUS = Australia; CA = Canada; CTI = cavotricuspid isthmus**;** LA = left atrium; PVI = pulmonary vein isolation; PWI = posterior wall isolation; RCT = randomized controlled trial; UK = United Kingdom; USA = United States of America.

∗ Data expressed as mean or median.

† Data from entire study.

Mean age of the patients was 62.7 years (range 52.7–68.5 years), and 76% (range 61%–84%) were male. One RCT included both paroxysmal and persistent AF.^20^ In this case, we only included data on patients with PeAF. Obesity and hypertension were the predominant comorbidities. Mean AF duration ranged from 12–64 months. Mean left atrial diameter was 44.5 mm (range 42–48.3 mm). Radiofrequency catheter ablation was predominantly used with only 2 RCTs utilizing cryoballoon ablation.^16^^,^^17^ Follow-up ranged from 10–23.8 months. During data collection, 4 authors were contacted for additional data, and 3 provided the information.

### Efficacy endpoints

**Figure 2:**
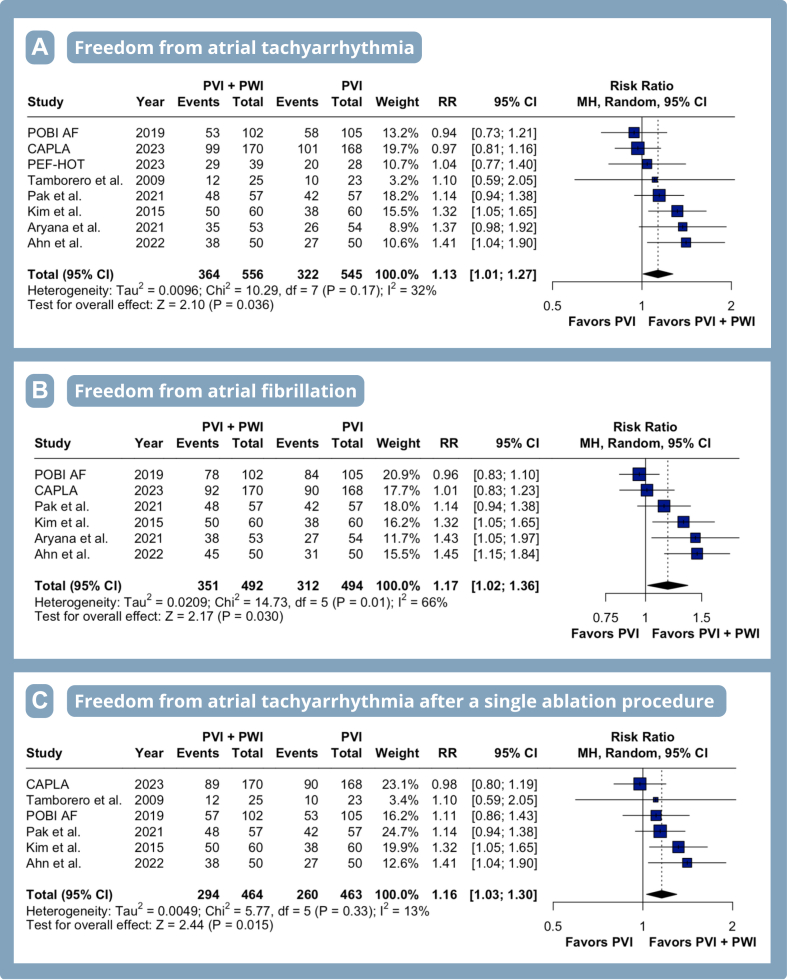
Meta-analysis of efficacy endpoints in patients with atrial fibrillation undergoing pulmonary vein isolation (PVI) with adjunctive posterior wall isolation (PWI). Forest plots presenting the risk ratio (RR) and 95% confidence interval (CI) for each treatment strategy on freedom from atrial tachyarrhythmia **(A),** freedom from atrial fibrillation **(B),** and freedom from atrial tachyarrhythmia after a single ablation procedure **(C)**. CI = confidence interval; MH = Mantel-Haenszel; RR = risk ratio.

PVI with adjunctive PWI overall resulted in a significantly higher rate of freedom from ATA (13% increase) compared with PVI alone (RR 1.13, 95% CI 1.01–1.27, *P* = .04, I^2^ = 32%) (Figure 2A), mainly due to improved freedom from AF (RR 1.17, 95% CI 1.02–1.36, *P* = .03, I^2^ = 66%) (Figure 2B). There was no statistical difference between groups for freedom from atrial flutter/tachycardia (RR 0.96, 95% CI 0.89–1.03, *P* = .22, I^2^ = 0%) (Supplemental Figure 1A).

**Figure 3:**
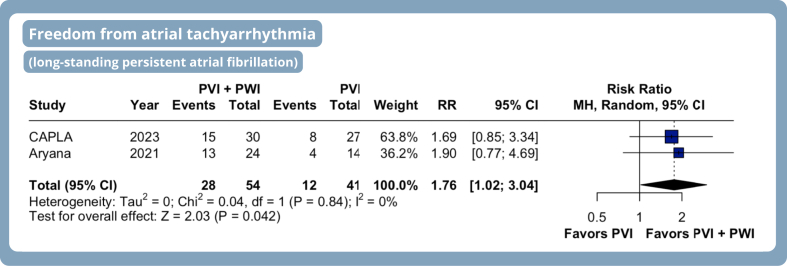
Meta-analysis of efficacy endpoints in patients with atrial fibrillation undergoing PVI with adjunctive PWI. Forest plots presenting RR and 95% CI for each treatment strategy on freedom from atrial tachyarrhythmia in patients with long-standing persistent atrial fibrillation. Abbreviations as in Figure 2.

Freedom from ATA after a single ablation procedure was significantly higher among patients receiving PVI with adjunctive PWI compared with PVI alone (RR 1.16, 95% CI 1.03–1.30, *P* = .02, I^2^ = 13%) (Figure 2C). Among patients with long-standing PeAF, adjunctive PWI resulted in a greater freedom from ATA (76% increase) compared with PVI alone (RR 1.76, 95% CI 1.02–3.04, *P* = .04, I^2^ = 0%) (Figure 3).

There was no significant difference between groups with regard to freedom from ATA without AAD (RR 1.12, 95% CI 0.99–1.27, *P* = .06, I^2^ = 21%) (Supplemental Figure 1B), freedom from ATA after a single procedure without AAD (RR 1.10, 95% CI 0.97–1.26, *P* = .14, I^2^ = 32%) (Supplemental Figure 1C) and need for cardioversion (RR 1.30, 95% CI 0.64–2.60, *P* = .47, I^2^ = 57%) (Supplemental Figure 1D). Also, the need for repeat ablation was not significantly different between groups (RR 1.38, 95% CI 0.63–3.01, *P* = .42, I^2^ = 34%) (Supplemental Figure 1E). In patients who received PVI with PWI, 19 of 29 patients (65.6%) exhibited posterior wall reconnection at repeat catheter ablation.

### Procedural endpoints

**Figure 4:**
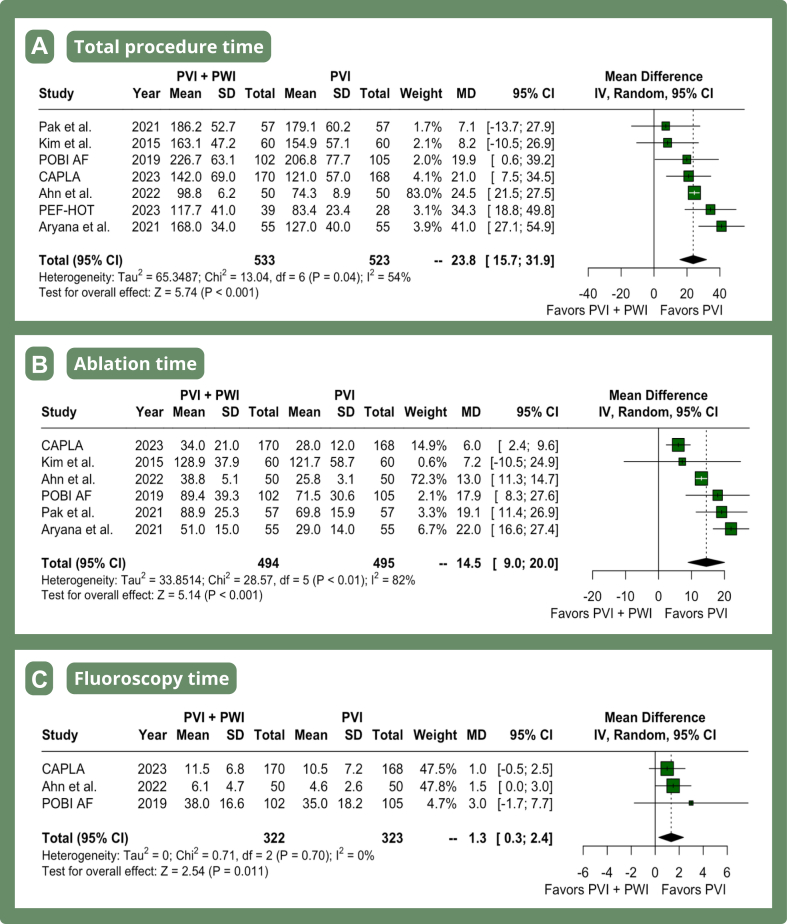
Meta-analysis of procedural endpoints in patients with atrial fibrillation undergoing PVI with adjunctive PWI. Forest plots presenting RR and 95% CI for each treatment strategy on total procedure time **(A),** ablation time **(B),** and fluoroscopy time **(C)**. IV = inverse variance; other abbreviations as in Figure 2.

PVI with adjunctive PWI was associated with a significantly higher total procedural time (MD 23.78 minutes, 95% CI 15.66–31.90 minutes, *P* <.01, I^2^ = 54%) (Figure 4A), ablation time (MD 14.50 minutes, 95% CI 8.97–20.02 minutes, *P* <.01, I^2^ = 82%) (Figure 4B), and fluoroscopy time (MD 1.33 minutes, 95% CI 0.30–2.36 minute3s, *P* = .01, I^2^ = 0%) (Figure 4C).

### Safety endpoints

There was no significant difference between groups concerning atrioesophageal fistula (RR 1.06, 95% CI 0.11–10.11, *P* = .96, I^2^ = 0%) (Supplemental Figure 2A), pericarditis (RR 0.78, 95% CI 0.15–4.03, *P* = .77, I^2^ = 0%) (Supplemental Figure 2B), cardiac tamponade (RR 1.36, 95% CI 0.27–6.93, *P* = .71, I^2^ = 5%) (Supplemental Figure 2C), and phrenic nerve injury (RR 1.29, 95% CI 0.28–5.93, *P* = .75, I^2^ = 0%) (Supplemental Figure 2D).

### Secondary endpoints

There was no significant difference between groups with regard to change in left atrial diameter (MD –1.07 mm, 95% CI –2.30 to 0.17 mm, *P* = .09, I^2^ = 0%) (Supplemental Figure 3A) and short-term (RR 0.79, 95% CI 0.62–1.00, *P* = .05, I^2^ = 0%) (Supplemental Figure 3B) and long-term (RR 0.80, 95% CI 0.64–1.01, *P* = .06, I^2^ = 0%) AAD therapy (Supplemental Figure 3C). However, these secondary outcomes likely are underpowered to rule out a significant difference between groups, given the fewer number of studies that reported on these results. Nonetheless, as seen, there were trend toward a benefit associated with PVI plus PWI.

### Subgroup and sensitivity analysis

**Figure 5:**
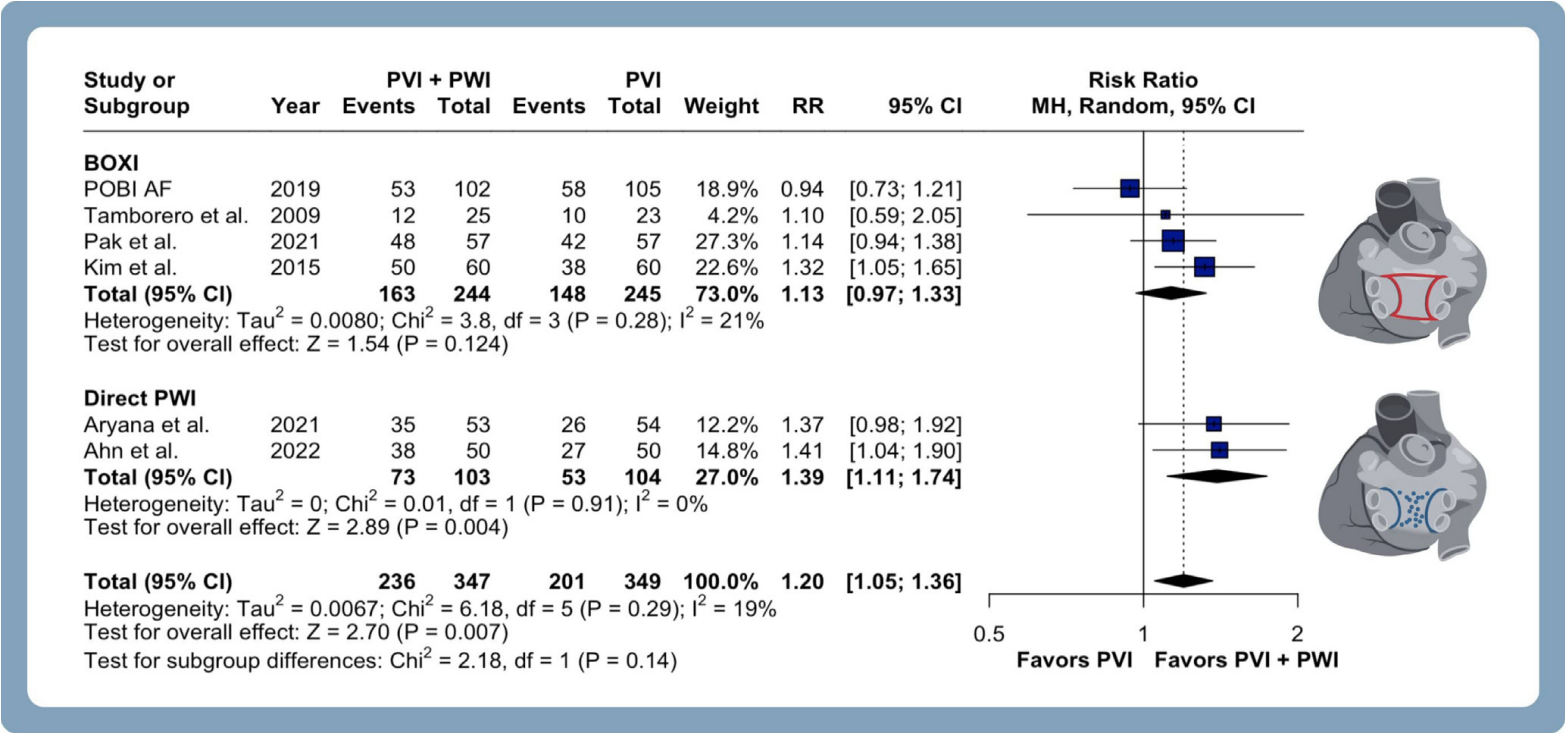
Subgroup analysis of the primary outcome in patients with atrial fibrillation undergoing PVI with adjunctive PWI stratified by PWI technique. Forest plots presenting RR and 95% CI for each subgroup strategy on freedom from atrial tachyarrhythmia recurrence stratified by (1) direct PWI vs (2) box isolation. BOXI = box isolation; other abbreviations as in Figure 2.

Freedom from ATA remained similar between PVI and PVI with adjunctive PWI when stratified by PWI technique (Figure 5), energy sources (Supplemental Figure 4A), overall risk of bias (Supplemental Figure 4B), and study location (Supplemental Figure 4C). After omitting studies performing an additional mitral isthmus ablation (RR 1.16, 95% CI 1.03–1.32, *P* = .015, I^2^ = 27%) (Supplemental Figure 5A), the results remained consistent. In an absolute risk assessment, PVI plus PWI increased freedom from ATA (RD 0.084, 95% CI 0.006–0.161, *P* = .035, I^2^ = 42%) (Supplemental Figure 5B), with an number needed to treat of 12.

### Meta-regression analysis

We performed meta-regression analyses to assess the impact of study-level characteristics and to explore potential sources of heterogeneity among the studies. We found no significant association between AF duration (*P* = .17) (Supplemental Figure 6A), mean age (*P* = .97) (Supplemental Figure 6B), left atrial diameter (*P* = .90) (Supplemental Figure 6C), or left ventricular ejection fraction (*P* = .06) (Supplemental Figure 6D) and the effectiveness of PVI with/without adjunctive PWI.

### Addressing heterogeneity

We conducted a graphic display of heterogeneity analysis to investigate the moderate to high heterogeneity in our findings. Our results were consistent across multiple simulations and remained stable after random exclusion of studies. A comprehensive explanation of statistical protocols to explore heterogeneity is available in Supplemental Results 1 and Supplemental Figures 7–9.

### Quality assessment

The RoB 2 results identified 5 RCTs with some concerns for bias^10^^,^^16^^,^^18^^,^^20^^,^^21^ and 3 at low risk of bias^9^^,^^17^^,^^19^ (Supplemental Figure 10). Funnel plot analysis and Egger regression test for the primary efficacy endpoint detected no evidence of publication bias for the included studies (*P* = .45) (Supplemental Figure 11).

## Discussion

This comprehensive meta-analysis of 8 RCTs enrolling 1104 patients examined the efficacy of PVI with adjunctive PWI in patients with PeAF. The main findings are as follows. First, PVI with adjunctive PWI was associated with a higher rate of freedom from ATA, primarily due to freedom from AF. Second, PVI with adjunctive PWI was also found to be superior to PVI alone for freedom from ATA after just a single ablation procedure. Third, the 2 strategies had no significant difference in the incidence of procedure-related adverse events. Fourth, in patients with long-standing PeAF, there was a greater benefit associated with adjunctive PWI regarding freedom from ATA.

Despite its superiority in maintaining sinus rhythm over AAD therapy, catheter ablation for the treatment of AF still has limited success rates, particularly in patients with PeAF.^1^^,^^2^ Previous clinical trials have reported single ablation procedure success rates of PVI in patients with PeAF ranging between 47% and 69% at 12 months, highlighting the suboptimal results of this technique in this challenging patient population.^22^^,^^23^ These results contrast with previously studied strategies, such as magnetic resonance imaging–guided ablation, complex fractionated electrogram ablation, and double wide-area circumferential ablation.^4^, ^5^, ^6^ The efficacy of PWI combined with PVI might stem from (1) the shared embryological origin of the posterior left atrial wall and the pulmonary veins;^8^ (2) the housing of the septopulmonary bundle within the posterior left atrium, a site of wavefront collision, facilitating formation of reentrant circuits^8^^,^^24^, ^25^, ^26^; and (3) the potential for structural remodeling in the left atrial posterior wall promoting AF substrates.^27^ Nevertheless, developing a personalized ablation strategy that targets individual patient substrates and AF triggers remains a challenge. Further studies are required to identify patients who could benefit from additional ablation strategies, with artificial intelligence potentially aiding in this selection process.

The RCTs included in our analysis did not verify the isolation of the posterior wall during long-term follow-up. However, 29 patients treated with PVI with PWI who experienced recurrent AF did undergo a repeat ablation, and approximately two-thirds exhibited posterior wall reconnection. Similarly, Bulava et al^28^ reported 77% PWI reconnection rate 3 months after radiofrequency catheter ablation. Because the cardiac autonomic nerves contribute to AF trigger mechanisms and are located on the subepicardial surface, it is believed that a transmural-plus lesion may be necessary during endocardial ablation.^29^^,^^30^ Hence, we can hypothesize that residual cardiac autonomic nerves could play a role in reconnection of the posterior wall and perhaps the recurrence of AF.

A subanalysis of patients with long-standing PeAF demonstrated a 79% increase in freedom from ATA with adjunctive PWI compared with PVI alone. This indicates that patients with longer AF durations may benefit from this additional procedure. Although our meta-regression analysis had not reached statistical significance, AF duration may be a significant variable affecting the relative effect of adjunctive PWI to PVI alone. Although the success rates of standard PVI decrease as the duration of AF increases, the addition of adjunctive PWI could have a greater effect on the outcomes of AF ablation. This could explain why the CAPLA and PEF-HOT trials had neutral findings, considering the short mean duration of AF reported in these studies (26.3 and 12 months, respectively).^9^^,^^10^

The subgroup analysis comparing direct posterior wall ablation vs posterior box isolation yielded intriguing findings (Figure 5). Pooled data from studies using posterior box isolation showed no significant difference in freedom from ATA (RR 1.13, 95% CI 0.97–1.33, I^2^ = 21%), whereas those using a direct posterior wall ablation approach demonstrated statistically significant results without heterogeneity (RR 1.39, 95% CI 1.11–1.74, I^2^ = 0%), despite no significant subgroup interaction (*P* = .14). It is important to mention that CAPLA and PEF-HOT trials were excluded from this subgroup analysis because their ablation strategy involved both direct posterior wall ablation and box isolation. These findings contribute to the ongoing discussion regarding the varying effectiveness of PWI techniques when used alongside PVI in the treatment of AF.^31^, ^32^, ^33^, ^34^ Direct posterior wall ablation could potentially offer increased reliability compared with box isolation.

Although previous meta-analyses also found a benefit of PVI with adjunctive PWI in reducing the recurrence of ATA in patients with both paroxysmal and persistent AF, in the current study, we decided to limit inclusion to patients with only PeAF for consistency and RCTs to minimize confounding variables in the analyses. Furthermore, we incorporated 4 additional RCTs (n = 553 patients), including the CAPLA trial, representing the largest multicenter RCT of PVI against PVI with PWI to date and a recent small RCT, the PEF-HOT trial. Moreover, additional endpoints and analyses were performed in our study, such as (1) the finding of superior efficacy of PVI with adjunctive PWI for freedom from ATA after a single ablation; and (2) a subanalysis showing a greater effect of PVI with PWI in patients with long-standing PeAF.

### Study limitations

First, the primary endpoint reported was based on the currently accepted standard of 30 seconds of arrhythmia recurrence, with a slight variation among the studies. AF burden recently has been increasingly considered a more clinically meaningful endpoint than the conventional recurrence definition. Nevertheless, the AF burden was only assessed in one of the included studies. Thus, it was not possible to perform a meta-analysis of AF burden as an endpoint. Second, there was moderate heterogeneity for the primary endpoint of freedom from ATA. However, we addressed increased heterogeneity by performing dedicated subgroup and meta-regression analyses, meticulously exploring the potential study-level and patient-level characteristics, as reported in the Supplemental Appendix. Third, the sample size in comparing subgroups and secondary endpoints is relatively small, potentially resulting in an underpowered analysis. Fourth, the studies did not assess the durability of PWI during follow-up in sinus rhythm, limiting our findings' mechanistic evaluation. Fifth, there were variations in the PVI and PWI techniques, given the operator’s preference, as outlined in Table 1. To mitigate that, we performed a sensitivity analysis omitting studies performing additional mitral line ablation, which showed consistent results with the main analysis.

## Conclusion

In this meta-analysis of RCTs, PVI with adjunctive PWI in patients with PeAF resulted in a higher rate of freedom from ATA, including AF, without an increase in procedure-related adverse events, compared with PVI alone. This benefit was substantially notable in patients with long-standing PeAF. These findings support the routine use of PWI as an adjunct strategy to PVI in patients with PeAF and longer AF durations.
