# Supplementary material for: Adjunctive posterior wall isolation for patients with persistent atrial fibrillation: A systematic review and meta-analysis
Source: Heart Rhythm O2. 2025 Jan 4;6(3):317–28. doi: 10.1016/j.hroo.2024.12.008 (PMC11973654; doi:10.1016/j.hroo.2024.12.008)
Supplement: Supplementary data [file mmc1.docx]

**SUPPLEMENTARY APPENDIX**

**Adjunctive Posterior Wall Isolation for Patients with Persistent Atrial Fibrillation: A Systematic Review and Meta-Analysis**

André Rivera,^1^ Antônio S. Menezes, MD, PhD,^2^ Douglas Mesadri Gewehr, MD,^3,4^ Bárbara Nascimento, MD,^5^ Isabele Ayumi Miyawaki,^6^ Luís E. Rohde, MD, PhD,^5^ Caique M. P. Ternes, MD,^5^ Arash Aryana, MD, PhD,^7^ André d’Avila, MD, PhD ^8^

*^1^Department of Medicine, Nove de Julho University, São Bernardo do Campo, Brazil*

*^2^Federal University of Goiás, Goiânia, Brazil*

*^3^Curitiba Heart Institute, Curitiba, Brazil*

*^4^Denton Cooley Institute of Research, Science, and Technology, Curitiba, Brazil*

*^5^Federal University of Rio Grande do Sul, Porto Alegre, Brazil*

*^6^Federal University of Paraná, Curitiba, Brazil*

*^7^Mercy General Hospital and Dignity Health Heart and Vascular Institute, Sacramento, California, USA*

*^8^Harvard Thorndike Electrophysiology Institute, Beth Israel Deaconess Medical Center, Boston, USA*

**Table of Contents**

[Supplemental Methods 1. PRISMA 2020 Main Checklist 3](#_Toc148895688)

[Supplemental Methods 2. Details of the Search Strategy 6](#_Toc148895689)

[Supplemental Methods 3. Enrollment Period by Study 7](#_Toc148895690)

[Supplemental Methods 4. Main Exclusion Criteria Used by Study 8](#_Toc148895691)

[Supplemental Methods 5. Methods Used to Confirm Isolation During Ablation 9](#_Toc148895692)

[Supplemental Methods 6. Endpoint Definitions and Methods used by Study 10](#_Toc148895693)

[Supplemental Methods 7. Addressing Heterogeneity 11](#_Toc148895694)

[Supplemental Results 1. Addressing Heterogeneity 12](#_Toc148895695)

[Supplemental Table 1. Clinical Baseline of the Patients Included in the Primary Analysis 14](#_Toc148895696)

[Supplemental Table 2. Baseline Patients’ Antiarrhythmic Drugs 15](#_Toc148895697)

[Supplemental Figure 1. Secondary Efficacy Endpoints 16](#_Toc148895698)

[Supplemental Figure 2. Safety Endpoints 18](#_Toc148895699)

[Supplemental Figure 3. Secondary Endpoints 20](#_Toc148895700)

[Supplemental Figure 4. Subgroup Analysis for the Primary Efficacy Endpoint 21](#_Toc148895701)

[Supplemental Figure 5. Sensitivity Analysis of the Primary Outcome 23](#_Toc148895702)

[Supplemental Figure 6. Meta-Regressions and Bubble Plots 24](#_Toc148895703)

[Supplemental Figure 7. Graphical Display of Study Heterogeneity (GOSH) 26](#_Toc148895704)

[Supplemental Figure 8. Baujat Plot for the Primary Efficacy Endpoint 30](#_Toc148895705)

[Supplemental Figure 9. Leave-one-out Analysis Sensitivity Analysis for the Primary Efficacy Endpoint 31](#_Toc148895706)

[Supplemental Figure 10. RoB 2 - Cochrane tool for assessing the risk of bias in randomized clinical trials. 32](#_Toc148895707)

[Supplemental Figure 11. Funnel Plot and Egger’s Test for the Primary Efficacy Endpoint 33](#_Toc148895708)

[Supplemental References 34](#_Toc148895709)

**Supplemental Methods 1.** PRISMA 2020 Main Checklist

| **Topic** | **No.** | **Item** | **Location where item is reported** |
| --- | --- | --- | --- |
| **TITLE** |  |  |  |
| **Title** | 1 | Identify the report as a systematic review. | Pg. 1 at MS |
| **ABSTRACT** |  |  |  |
| **Abstract** | 2 | See the PRISMA 2020 for Abstracts checklist | NA |
| **INTRODUCTION** |  |  |  |
| **Rationale** | 3 | Describe the rationale for the review in the context of existing knowledge. | Pg. 4 at MS |
| **Objectives** | 4 | Provide an explicit statement of the objective(s) or question(s) the review addresses. | Pg. 4 at MS |
| **METHODS** |  |  |  |
| **Eligibility criteria** | 5 | Specify the inclusion and exclusion criteria for the review and how studies were grouped for the syntheses. | Pg 5-8 at MS |
| **Information sources** | 6 | Specify all databases, registers, websites, organisations, reference lists and other sources searched or consulted to identify studies. Specify the date when each source was last searched or consulted. | Pg. 5 at MS |
| **Search strategy** | 7 | Present the full search strategies for all databases, registers and websites, including any filters and limits used. | Pg. 6 at sup |
| **Selection process** | 8 | Specify the methods used to decide whether a study met the inclusion criteria of the review, including how many reviewers screened each record and each report retrieved, whether they worked independently, and if applicable, details of automation tools used in the process. | Pg. 5-6 at MS |
| **Data collection process** | 9 | Specify the methods used to collect data from reports, including how many reviewers collected data from each report, whether they worked independently, any processes for obtaining or confirming data from study investigators, and if applicable, details of automation tools used in the process. | Pg. 6 at MS |
| **Data items** | 10a | List and define all outcomes for which data were sought. Specify whether all results that were compatible with each outcome domain in each study were sought (e.g. for all measures, time points, analyses), and if not, the methods used to decide which results to collect. | Pg. 6-7 at MS |
|  | 10b | List and define all other variables for which data were sought (e.g. participant and intervention characteristics, funding sources). Describe any assumptions made about any missing or unclear information. | Pg. 6 at MS |
| **Study risk of bias assessment** | 11 | Specify the methods used to assess risk of bias in the included studies, including details of the tool(s) used, how many reviewers assessed each study and whether they worked independently, and if applicable, details of automation tools used in the process. | Pg. 7 at MS |
| **Effect measures** | 12 | Specify for each outcome the effect measure(s) (e.g. risk ratio, mean difference) used in the synthesis or presentation of results. | Pg 7 at MS |
| **Synthesis methods** | 13a | Describe the processes used to decide which studies were eligible for each synthesis (e.g. tabulating the study intervention characteristics and comparing against the planned groups for each synthesis (item 5)). | Table 1 |
|  | 13b | Describe any methods required to prepare the data for presentation or synthesis, such as handling of missing summary statistics, or data conversions. | Pg. 7-8 at MS |
|  | 13c | Describe any methods used to tabulate or visually display results of individual studies and syntheses. | Pg. 8 at MS, Pg. 12-13 at sup. |
|  | 13d | Describe any methods used to synthesize results and provide a rationale for the choice(s). If meta-analysis was performed, describe the model(s), method(s) to identify the presence and extent of statistical heterogeneity, and software package(s) used. | Pg. 7-8 at MS, Pg. 12-13 at sup. |
|  | 13e | Describe any methods used to explore possible causes of heterogeneity among study results (e.g. subgroup analysis, meta-regression). | Pg. 7-8 at MS |
|  | 13f | Describe any sensitivity analyses conducted to assess robustness of the synthesized results. | Pg. 12-13 at sup. |
| **Reporting bias assessment** | 14 | Describe any methods used to assess risk of bias due to missing results in a synthesis (arising from reporting biases). | Pg. 7 at MS |
| **Certainty assessment** | 15 | Describe any methods used to assess certainty (or confidence) in the body of evidence for an outcome. | NA |
| **RESULTS** |  |  |  |
| **Study selection** | 16a | Describe the results of the search and selection process, from the number of records identified in the search to the number of studies included in the review, ideally using a flow diagram. | Figure 1 |
|  | 16b | Cite studies that might appear to meet the inclusion criteria, but which were excluded, and explain why they were excluded. | NA |
| **Study characteristics** | 17 | Cite each included study and present its characteristics. | Table 1, Pg. 7-10, 14-15 at sup. |
| **Risk of bias in studies** | 18 | Present assessments of risk of bias for each included study. | Pg. 11 at MS, Pg. 29 at sup. |
| **Results of individual studies** | 19 | For all outcomes, present, for each study: (a) summary statistics for each group (where appropriate) and (b) an effect estimates and its precision (e.g. confidence/credible interval), ideally using structured tables or plots. | Fig. 2-4 at MS, Pg. 16-18 at sup. |
| **Results of syntheses** | 20a | For each synthesis, briefly summarise the characteristics and risk of bias among contributing studies. | Pg. 11 at MS, Pg. 19 at sup. |
|  | 20b | Present results of all statistical syntheses conducted. If meta-analysis was done, present for each the summary estimate and its precision (e.g. confidence/credible interval) and measures of statistical heterogeneity. If comparing groups, describe the direction of the effect. | Pg 9-10 at MS |
|  | 20c | Present results of all investigations of possible causes of heterogeneity among study results. | Pg. 11 at MS, Pg. 12-13 at sup. |
|  | 20d | Present results of all sensitivity analyses conducted to assess the robustness of the synthesized results. | Pg. 12-13 at sup. |
| **Reporting biases** | 21 | Present assessments of risk of bias due to missing results (arising from reporting biases) for each synthesis assessed. | Pg. 29-30 at sup. |
| **Certainty of evidence** | 22 | Present assessments of certainty (or confidence) in the body of evidence for each outcome assessed. | NA |
| **DISCUSSION** |  |  |  |
| **Discussion** | 23a | Provide a general interpretation of the results in the context of other evidence. | Pg. 12-14 at MS |
|  | 23b | Discuss any limitations of the evidence included in the review. | Pg. 14 at MS |
|  | 23c | Discuss any limitations of the review processes used. | Pg. 14 at MS |
|  | 23d | Discuss implications of the results for practice, policy, and future research. | Pg. 15 at MS |
| **OTHER INFORMATION** |  |  |  |
| **Registration and protocol** | 24a | Provide registration information for the review, including register name and registration number, or state that the review was not registered. | PROSPERO; CRD42023397998 |
|  | 24b | Indicate where the review protocol can be accessed, or state that a protocol was not prepared. | https://www.crd.york.ac.uk/prospero/display_record.php?RecordID=397998 |
|  | 24c | Describe and explain any amendments to information provided at registration or in the protocol. | More outcomes were available and main outcome was changed. |
| **Support** | 25 | Describe sources of financial or non-financial support for the review, and the role of the funders or sponsors in the review. | None |
| **Competing interests** | 26 | Declare any competing interests of review authors. | Pg. 1 |
| **Availability of data, code and other materials** | 27 | Report which of the following are publicly available and where they can be found: template data collection forms; data extracted from included studies; data used for all analyses; analytic code; any other materials used in the review. | Non-Available |

^Abbreviations: MS, manuscript; sup., supplement.^

# Supplemental Methods 2. Details of the Search Strategy

| **Search Strategy for all databases** |
| --- |
| (“atrial fibrillation” OR AF OR A-fib OR Afib) **AND** (PWI OR “posterior wall” OR “posterior left atrial wall” OR “posterior LA wall” OR “LA posterior wall” OR “left atrial posterior wall” OR “posterior left atrium” OR box OR “posterior box” OR “single ring” OR debulking) **AND** (PVI OR pulmonary vein) **AND** (ablation OR isolation) |

# Supplemental Methods 3. Enrollment Period by Study

| **Study** | **Enrollment Period** |
| --- | --- |
| Kistler, 2023  (CAPLA) | July 2018 - March 2021 |
| Wong, 2023  (PEF-HOT) | NA |
| Ahn, 2022 | December 2019 - July 2020 |
| Aryana, 2021 | February 2017 – April 2019 |
| Pak, 2021  (PEACEFUL) | June 2014 - ongoing |
| Lee, 2019  (POBI-AF) | March 2016 – January 2019 |
| Yu, 2017 | June 2014 - ongoing |
| Kim, 2015 | January 11 – August 2012 |
| Tamborero, 2009 | NA |

# Supplemental Methods 4. Main Exclusion Criteria Used by Study

| **Study and year** | **Main exclusion criteria** |
| --- | --- |
| Kistler, 2023  (CAPLA) | Long-standing persistent AF > 3 years; AF secondary to reversible cause; severe valvular heart disease or cyanotic; congenital heart disease; hypertrophic cardiomyopathy |
| Wong, 2023  (PEF-HOT) | Long-standing persistent AF > 3 years and LAD > 60 mm |
| Ahn, 2022 | Prior AF ablation or cardiac surgery; CKD with clearance rate <30mL/min |
| Aryana, 2021 | AF with a reversible cause; prior LA ablation; LVEF < 40%; LAD > 50 mm; prior MI; congenital heart disease; hypertrophic cardiomyopathy; LVEF < 40%; class IV HF |
| Pak, 2021  (PEACEFUL) | AF with rheumatic valvular disease; LAD ≥ 55 mm; prior AF ablation or cardiac surgery; valvular AF; structural heart disease |
| Lee, 2019  (POBI-AF) | AF with rheumatic valvular disease; LAD ≥ 60 mm; prior AF ablation or cardiac surgery; valvular AF |
| Kim, 2015 | Prior AF ablation or cardiac surgery, cardiomyopathy, or congenital heart disease |
| Tamborero, 2009 | Prior AF ablation |
| LVEF, left ventricular ejection fraction; HF, heart failure; CKD, chronic kidney disease; LAD, left atrial diameter; MI, myocardial infarction. | |

# Supplemental Methods 5. Methods Used to Confirm Isolation During Ablation

| **Study** | **Methods used for ablation confirmation** | |
| --- | --- | --- |
| Kistler, 2023  (CAPLA) | Presence of entrance and exit block, along with the presence of spontaneous potentials or the complete absence of local electrograms without any response to high-output pacing. | |
| Wong, 2023  (PEF-HOT) | Presence of entrance block (noise floor 0.04 mV) and exit block pacing at 20 mA at 2 milliseconds | |
| Ahn, 2022 | Confirmed by high-density voltage mapping. | |
| Aryana, 2021 | A detailed post-ablation 3-dimensional electroanatomic map was recreated in each patient using the high-density mapping catheter to confirm PVI or PVI with PWI, as applicable (cutoff 0.10 to 0.50 mV). In addition to detailed voltage mapping in sinus rhythm, PVI and PVI with PWI were confirmed using high-output pacing (>10 mA) within the PVs to test for entrance or exit block before and after intravenous drug stimulation (adenosine). Pacing maneuvers to confirm PWI were also performed extensively from multiple sites within the area of isolation (the PV component) before and after intravenous drug stimulation. | |
| Pak, 2021  (PEACEFUL) | For PVI, electrical isolation of PV potentials and bidirectional block were confirmed. | For PWI, successful bidirectional block of the roof line, (2) voltage abatement of <0.1 mV in the LA posterior wall, and (3) entrance and exit block. |
| Lee, 2019  (POBI-AF) | For PVI, verified during an isoproterenol infusion after a 30-min waiting time. | For PWI, 1) successful bidirectional block of the roof line; 2) voltage abatement of <0.1 mV in the LA posterior wall; and 3) entrance and exit block. |
| Yu, 2017 | For PVI, electric isolation of PV potentials and bidirectional block of PVs. | For PWI, no endocardial electrogram in the LA posterior wall with a roof line block. |
| Kim, 2015 | For PVI differential pacing maneuver on either side of the linear lesions after the restoration of sinus rhythm. | For PWI, defined as the absence of local potentials or dissociation potentials in the posterior wall of the LA and lack of LA capture by pacing maneuver. |
| Tamborero, 2009 | For PVI, local electrogram inside the encircled area disappeared or was dissociated or, when this was not possible, until the bipolar voltage amplitude dropped to 0.15 mV; electric block was confirmed by the inability to conduct to the LA after pacing at several sites within the PV antrum. | For PWI, confirmed by the inability to conduct to the remaining atria after pacing at several sites within the surrounded LA posterior region with the ablation catheter, observing the local capture in the proximal bipole of the pacing catheter when possible. |

# Supplemental Methods 6. Endpoint Definitions and Methods used by Study

| **Study** | **Atrial tachyarrhythmia (ATA)** | **Method** | **Clinical ATA recurrence** |
| --- | --- | --- | --- |
| Kistler, 2023  (CAPLA) | AF, AFL, or AT | ILR or CIED: 53 (15.7%)  Frequent ECG monitoring: 237(70.1%)  24-h Holter each visit (3, 6, 9, and 12 months): 47 (13.9%) | ATA lasting > 30 seconds and occurring after a 3-month blanking period after a single ablation procedure. |
| Wong, 2023  (PEF-HOT) | AF, AFL, or AT | Follow-up at 1, 3, 6, and 12 months, with 2-week continuous ECG monitoring at 3 and 12 months |  |
| Ahn, 2022 | AF, AFL, or AT | ECG each visit and 24h-Holter at 3, 6, and 12 months  ECG whenever a patient presented with palpitations |  |
| Aryana, 2021 | AF, AFL, or AT | ECG each visit. Mobile cardiac telemetry at 3-, 6-, and 9-months post-ablation unless pre-existing CIED |  |
| Pak, 2021  (PEACEFUL) | AF or AFL | ECG each visit. 24-hour Holter at 3 and 6 months, then every 6 months |  |
| Lee, 2019  (POBI-AF) | AF or AFL | ECG each visit. 24-hour Holter at 3 and 6 months, then every 6 months |  |
| Yu, 2017 | AF or AFL | ECG each visit. 24-hour Holter at 3 and 6 months, then every 6 months |  |
| Kim, 2015 | AF or AFL | ECG each visit and whenever patients reported palpitations. 48-hour Holter at 1-, 3-, 6- and 12-months post-ablation | Any patient with documented AF or atrial flutter (AFL) during the follow-up period was diagnosed as having a clinical arrhythmia recurrence. |
| Tamborero, 2009 | AF or AFL | 48-hour Holter before each visit at 1, 4, and 7 months, then every 6 months if asymptomatic | AF recurrences or LA flutter after a blanking period of 3 months. |
| Abbreviations: ATA, atrial tachyarrhythmia; AF, atrial fibrillation; AFL, atrial flutter; AT, atrial tachycardia; ILR, implantable loop recorder; CIED, cardiac implantable electronic device; ECG, electrocardiography; LA, left atrium | | | |

# Supplemental Methods 7. Addressing Heterogeneity

To ensure the reliability and robustness of our findings, we conducted a thorough analysis to identify potential outliers and influential studies for the primary outcome. This included the use of a Graphic Display of Heterogeneity (GOSH) analysis, in which we performed multiple random simulations and exclusions. The GOSH plot was generated, and three unsupervised machine learning (ML) algorithms were applied to detect clusters in the data, namely the k-means algorithm, density-based spatial clustering of applications with noise (DBSCAN), and Gaussian mixture models.

We created a Baujat plot and performed a leave-one-out sensitivity analysis to investigate potential outliers for the primary efficacy endpoint. The leave-one-out sensitivity analysis removed one study at a time to ensure that our results were not reliant on a single study. These methods helped to identify any potential outliers.

It is worth noting that the MH default continuity correction of 0.5 (default behavior in metabin function [R meta package]) is only necessary when one specific cell is zero in all included studies in the meta-analysis. However, its use in other scenarios has been discouraged by Efthimiou et al. [7], as it can lead to biased results. Therefore, we only applied the continuity correction in the specific situation mentioned above, and when it was not fitted, we used the exact MH method without continuity correction.

# Supplemental Results 1. Addressing Heterogeneity

Hence, due to the moderate heterogeneity of the results, we performed Graphical Display of Study Heterogeneity (GOSH) analyses for the primary endpoint. The GOSH plot illustrates the effect size plotted against the *I^2^* for all possible combinations of studies. The 255 possible subsets of meta-analysis (2^k^ – 1 possible combinations) for freedom from atrial tachyarrhythmia recurrence (ATA) are presented as a GOSH plot in Supplementary Figure 7A. By analyzing the pattern in our data, we find that most values are concentrated in a cluster with high heterogeneity and a symmetrical distribution along the overall estimate axis. The distribution of *I^2^* is relatively bimodal, with clusters following a moderate *I^2^* sparse and considerable number of study combinations for which the estimated heterogeneity was null.

To find out which studies cause this shape, we applied three unsupervised machine learning (ML) algorithms, detailed in methods, to detect clusters in the GOSH plot data (Supplemental Figures 7B-D*)*. Ultimately, one potential outlier was identified similarly by the three unsupervised ML algorithms. The corresponding subset, including this potential outlier, is demonstrated in Supplemental Figure 7E-F. In summary, the GOSH analysis showed that heterogeneity significantly changed when the CAPLA trial was excluded from the analysis. The overall effect did not change significantly before and after excluding random studies in the GOSH plot. However, results also show that the results are stable on multiple simulations, despite significant heterogeneity.

We further explored each study`s influence by performing a Baujat plot leave-one-out sensitivity analysis (Supplemental Figure 8) and plotting the leave-one-out sensitivity analysis (Supplementary Figure 9). The analyses showed that most of the heterogeneity was carried out by CAPLA trial, as already identified by GOSH analysis. By inspecting the leave-one-out sensitivity analysis, when the CAPLA trial is omitted, the pooled effect estimates (RR) varied from 1.13 to 1.18, accompanied by a significant decrease of heterogeneity (*I^2^*) from 32% to 13%. The CAPLA trial had the second smallest AF duration period and the largest sample size, contributing to the heterogeneity in the meta-analytic summary estimates.

# Supplemental Table 1. Clinical Baseline of the Patients Included in the Primary Analysis

| **Study** | **Patients**  **PWI (-)/PWI(+)** | **Age^†^ , y** | **Male, n (%)** | **AF duration** †**, months** | **LVEF^†^, %** | **LAD^†^, mm** | **HTN, n (%)** | **HF, n (%)** | **CHA2DS2-VASc score**† | **Follow-up^†^ (months)** |
| --- | --- | --- | --- | --- | --- | --- | --- | --- | --- | --- |
| Kistler, 2023  (CAPLA) | 168/170 | 65.6 | 259 (76%) | 26.29 | 55.5 | 45 | 159 (47%) | 98 (29%) | 2 | 12 |
| Wong, 2023  (PEF-HOT) | 28/39 | 68.4 | 51 (76.1) | 8.4 (3–25) | 52.1 | 47 | 49 (73.1) | 21 (31.3%) | 2.7 | 12.4 |
| Ahn, 2022 | 50/50 | 65.5 | 84 (84%) | 56.15 | 58 | 48.3 | 83 (83%) | 45 (45%) | 3 | 15 |
| Aryana, 2021 | 55/55 | 68.5 | 68 (61.8%) | NA | 60.5 | 44 | 62 (53.3%) | 29 (26.4%) | 2.6 | 21 |
| Pak, 2021  (PEACEFUL) | 57/57 | 60.1 | 82 (71.9%) | 24 (10–60) | 60.5 | 42 | 58 (50.9%) | 23 (20.2%) | 2 | 23.8 |
| Lee, 2019  (POBI-AF) | 105/102 | 58.7 | 172 (83.1%) | 38.5 ± 38.8 | 59 | 44.8 | 97 (46.9%) | 47 (22.7%) | 1.72 | 16.2 |
| Yu, 2017 | 59/54 | 60.4 | 85 (75%) | 42.8 ± 44.4 | 62 | 42.7 | 28 (52%) | 19 (17%) | 2.2 | 18.6 |
| Kim, 2015 | 60/60 | 57.2 | 87 (72.5%) | NA | 63.7 | 42.2 | 54 (45%) | 23 (19.2%) | NA | 12 |
| Tamborero, 2009 | 23/25 | 52.7* | 92* (76.7%) | 63.9* | 59.6* | 41.3* | 55* (45.8%) | NA | NA | 10±4 |

*Data from the entire study population, not just in persistent atrial fibrillation;  †Mean or median; AAD: anti-arrhythmic drugs; LSP-AF, long-standing persistent atrial fibrillation; HTN, hypertension; HF, heart failure; PWI, posterior wall isolation; LAD, left atrial diameter; LVEF, left ventricular ejection fraction; CHA2DS2-VASC, congestive heart failure, hypertension, age ≥75 (doubled), diabetes, stroke (doubled), vascular disease, age 65 to 74 and sex category (female)

# Supplemental Table 2. Baseline Patients’ Antiarrhythmic Drugs

| **Study** | **Class 1** | **Class 3** |
| --- | --- | --- |
| Kistler, 2023  (CAPLA) | 48 (14.2%) | 186 (55%) |
| Wong, 2023  (PEF-HOT) | NA | |
| Ahn, 2022 | 36 (36%) | 64 (64%) |
| Aryana, 2021 | 35(31.8%) | |
| Pak, 2021  (PEACEFUL) | 47 (41.2%) | 67 (58.8%) |
| Lee, 2019  (POBI-AF) | NA | |
| Yu, 2017 | 64 (56.6%) | 49 (43.3%) |
| Kim, 2015 | NA | |
| Tamborero, 2009 |  |  |

# Supplemental Figure 1. Secondary Efficacy Endpoints

**Figure 1A.** Freedom from Atrial Flutter/Tachycardia

**
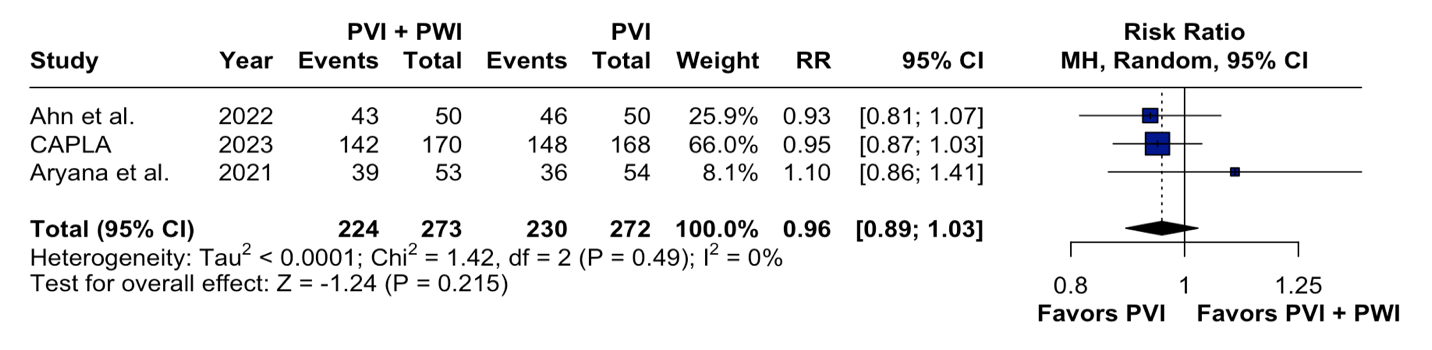
**

**Figure 1B.** Freedom from ATA without AAD


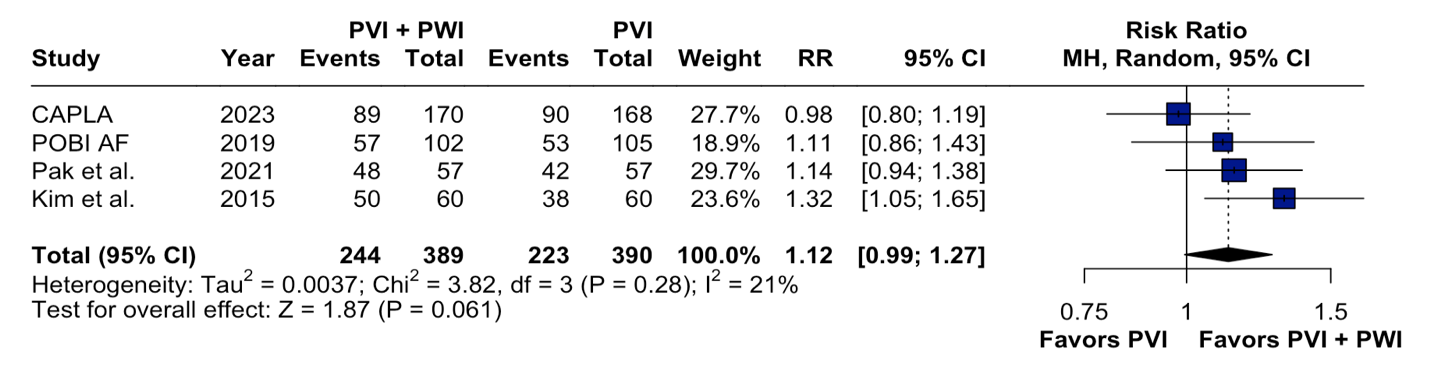


**Figure 1C.** Freedom from ATA after a Single Procedure without AAD


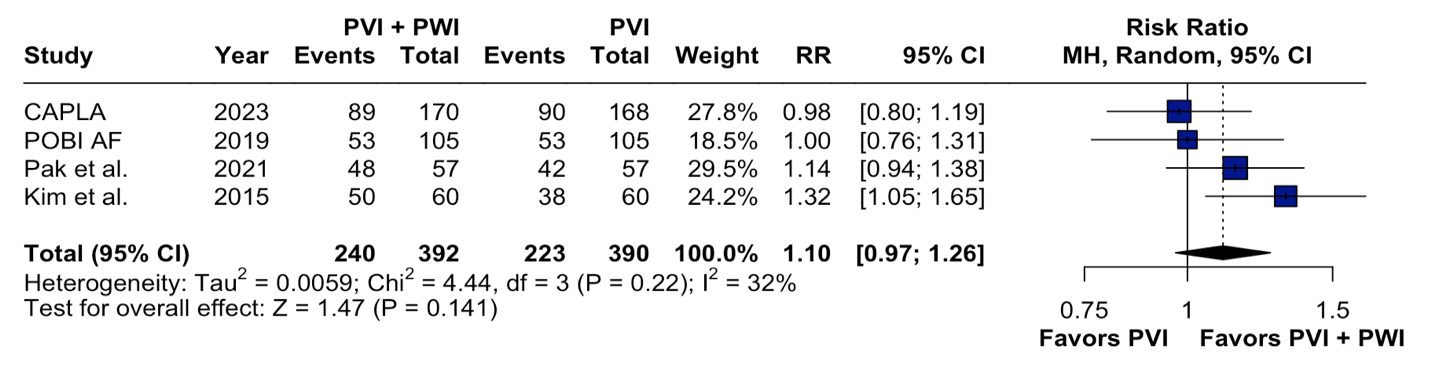


**Figure 1D.** Need for Cardioversion


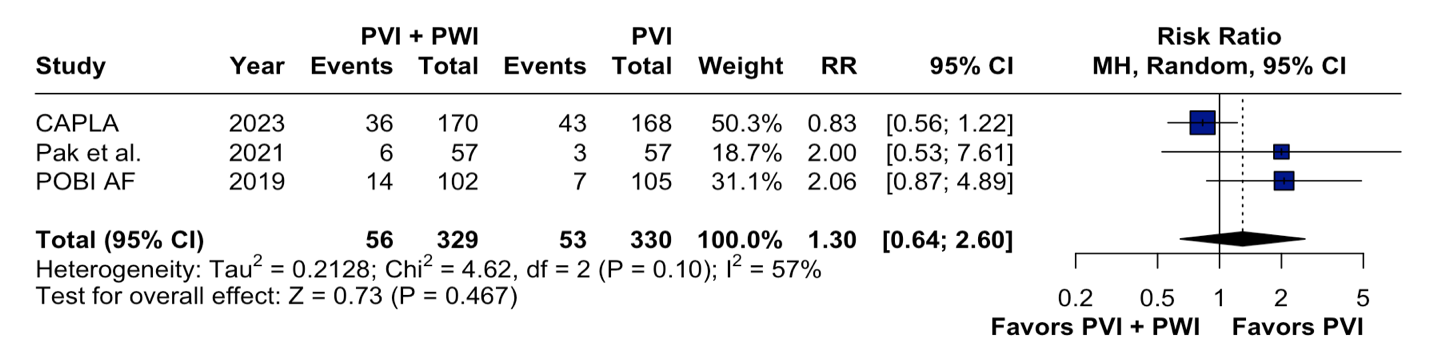


**Figure 1E.** Need for Repeated Ablation


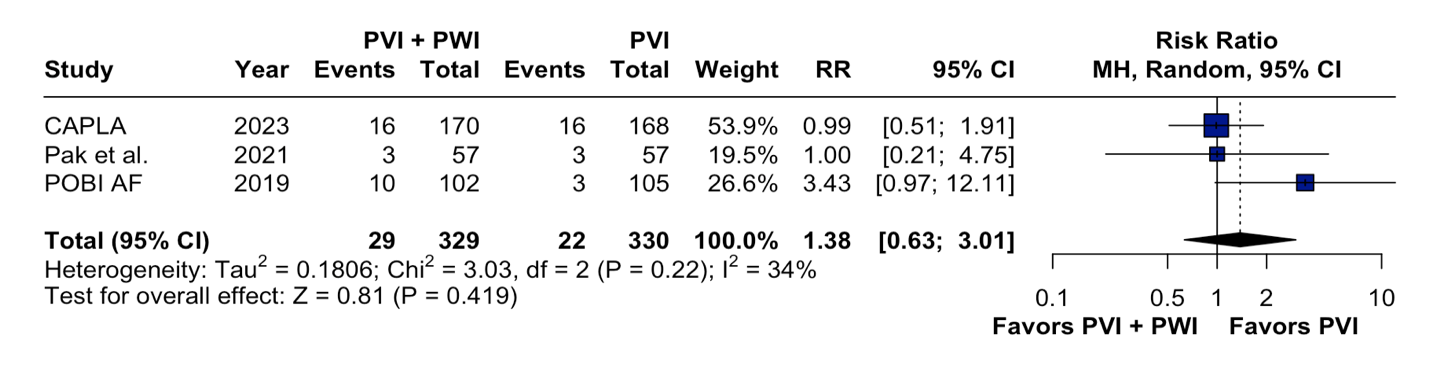


# Supplemental Figure 2. Safety Endpoints

**Figure 2A.** Atrioesophageal fistula


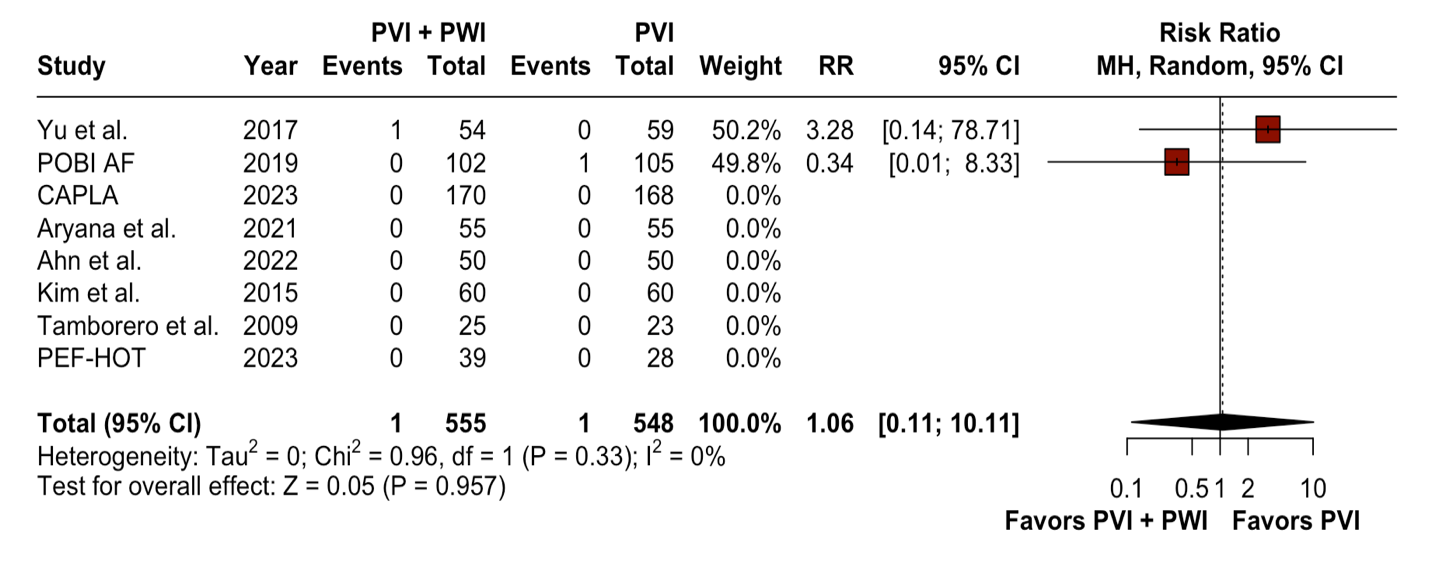


**Figure 2B.** Pericarditis

**
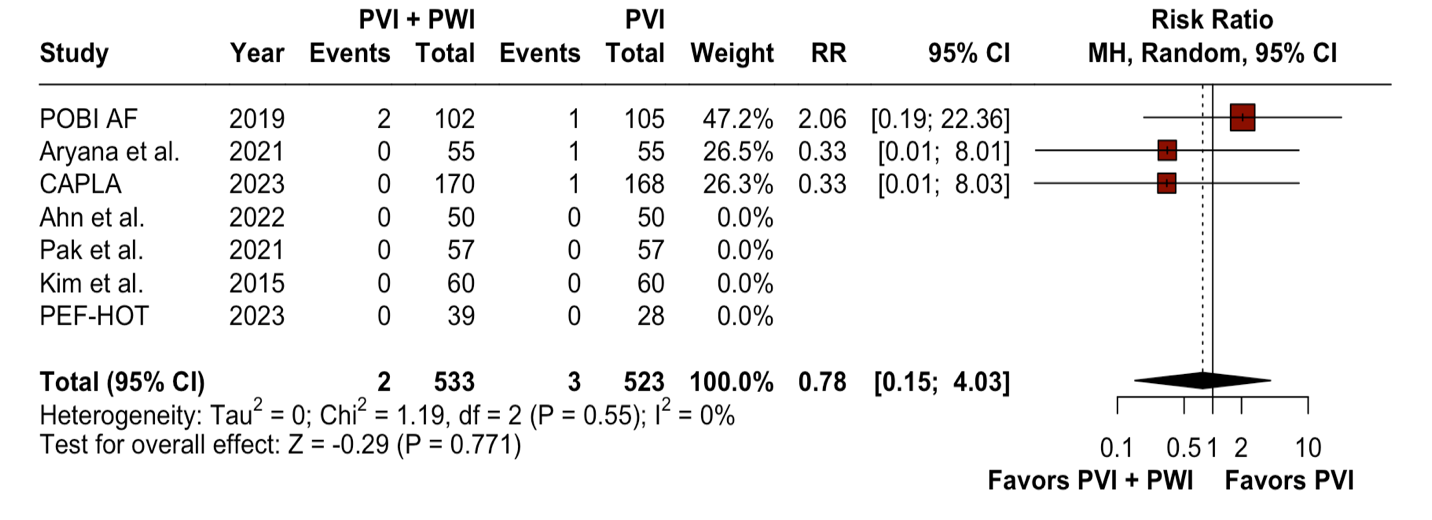
**

**Figure 2C.** Cardiac tamponade

**
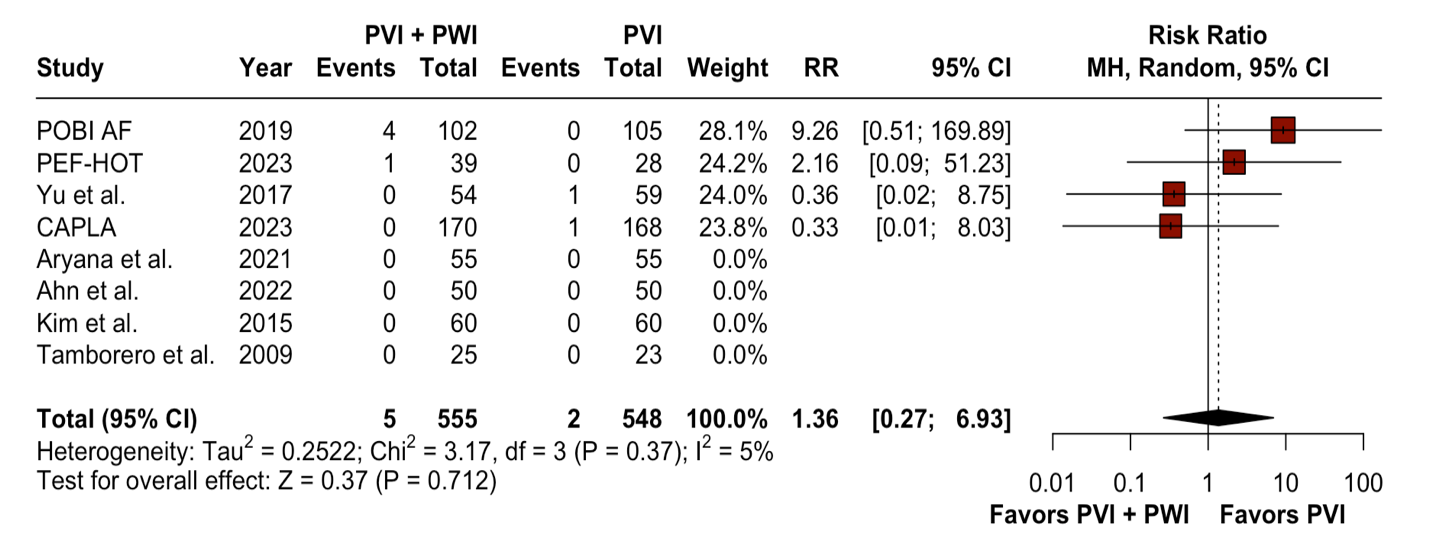
**

**Figure 2D.** Phrenic nerve injury


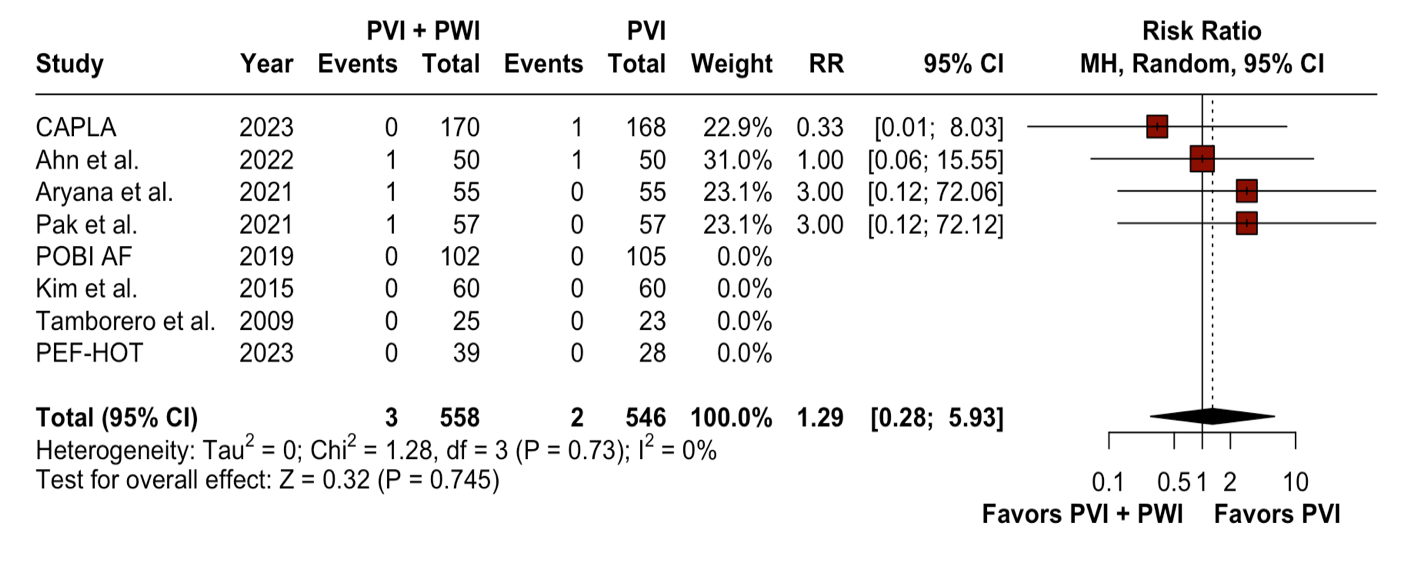


# Supplemental Figure 3. Secondary Endpoints

**Figure 3A.** Left atrial diameter change (mm)


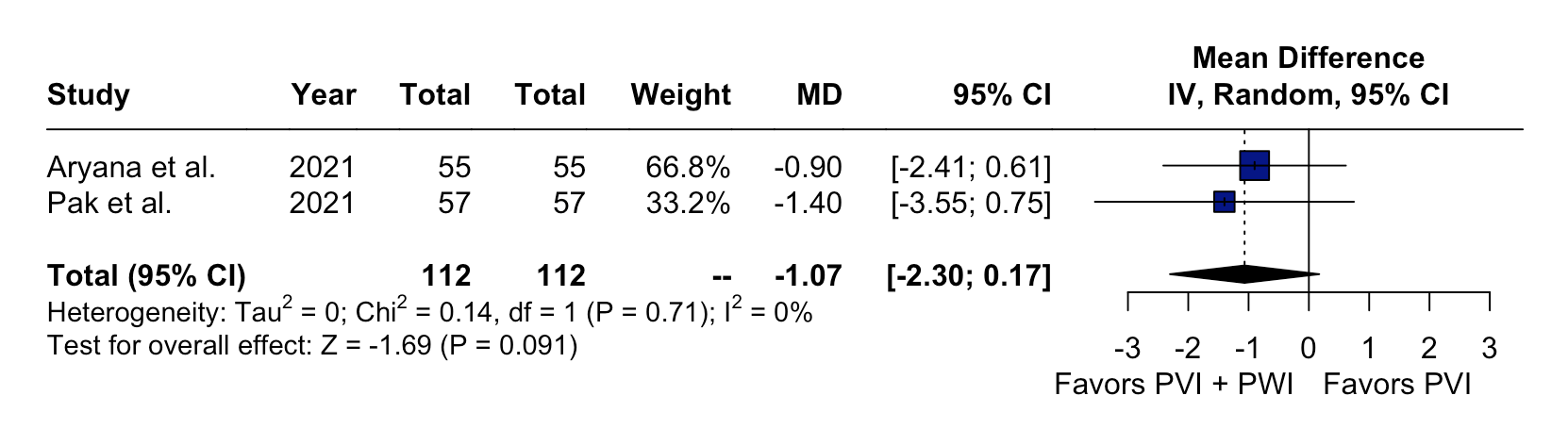


**Figure 3B.** Short-term AAD prescription (<12 months)


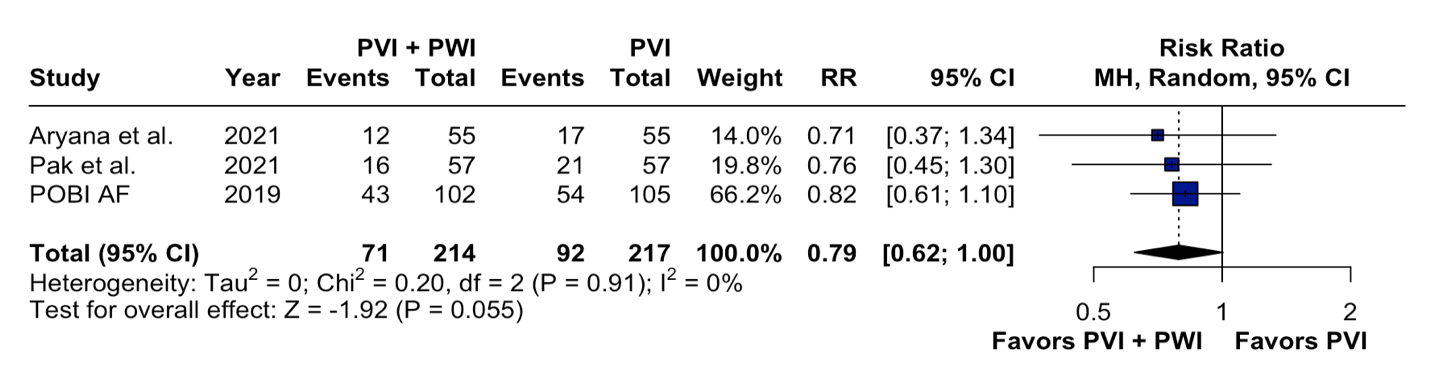


**Figure 3C.** Long-term AAD prescription (≥12 months)


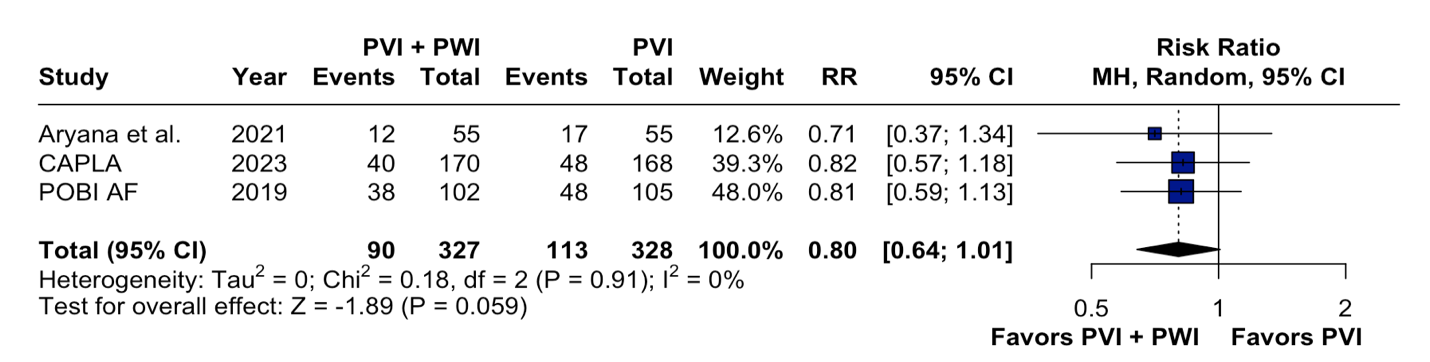


# Supplemental Figure 4. Subgroup Analysis for the Primary Efficacy Endpoint

**Figure 4A.** Primary Efficacy Endpoint Stratified by Energy Source Used.


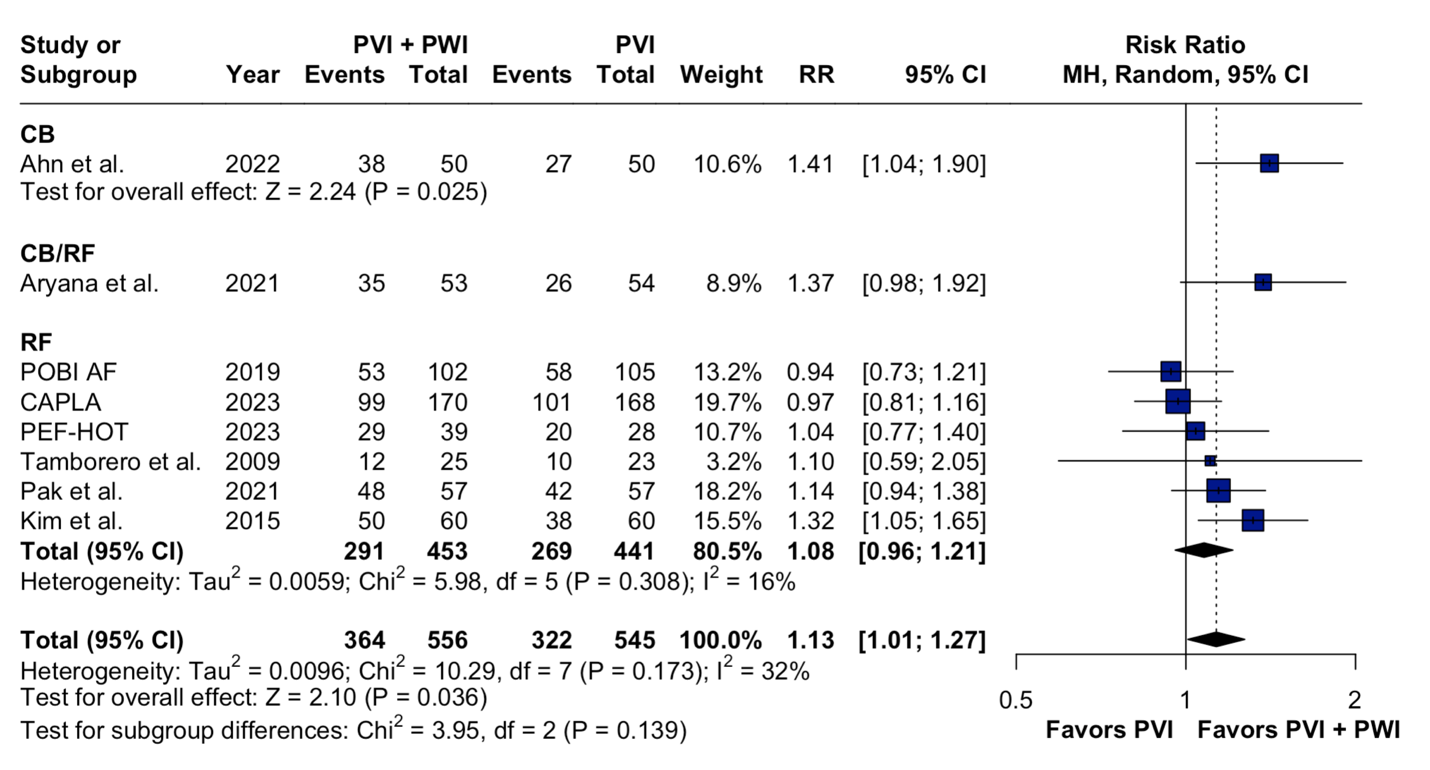


**Figure 4B.** Primary Efficacy Endpoint Stratified by Overall Risk of Bias.


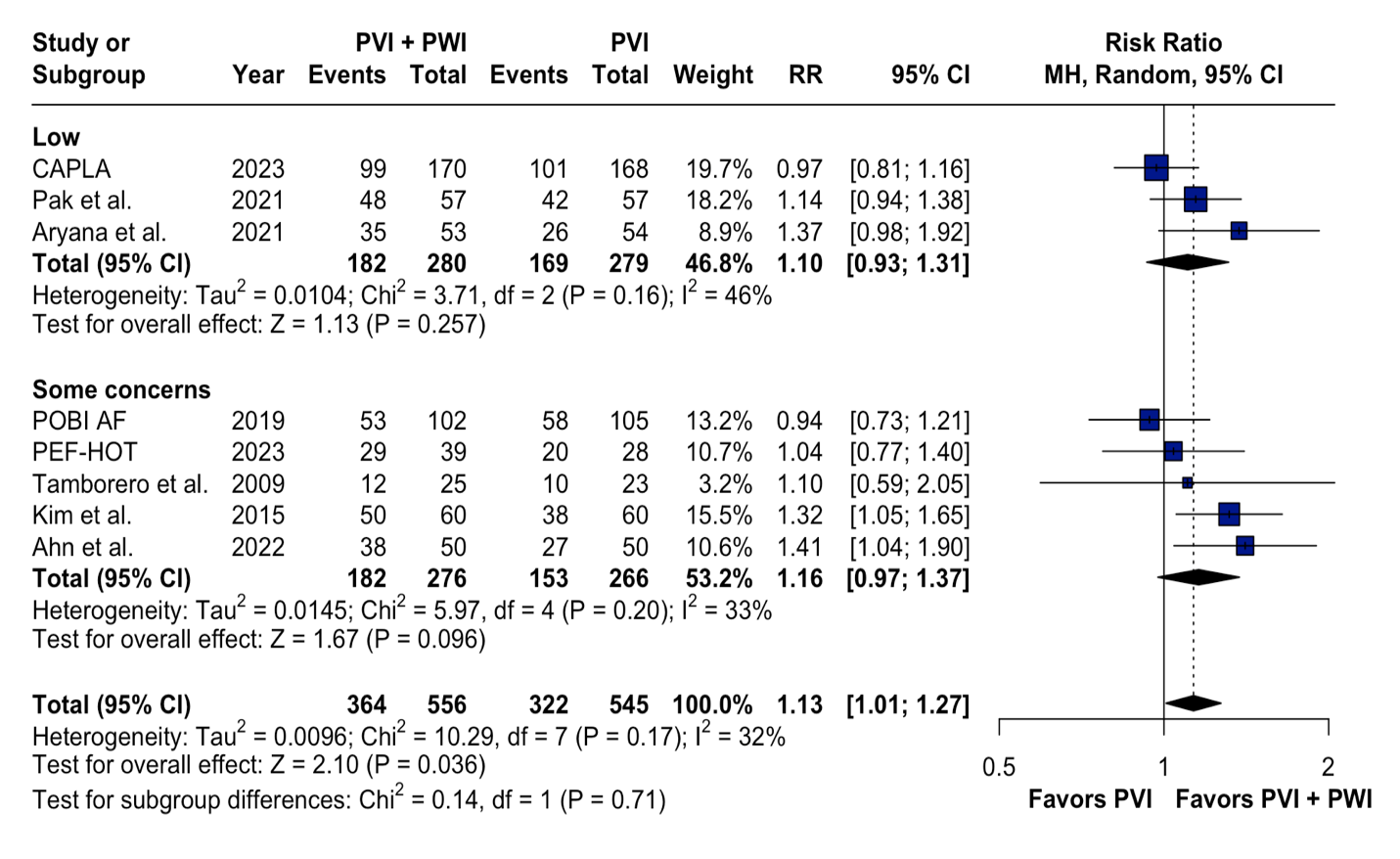


**Figure 4C.** Primary Efficacy Endpoint Stratified by Country.


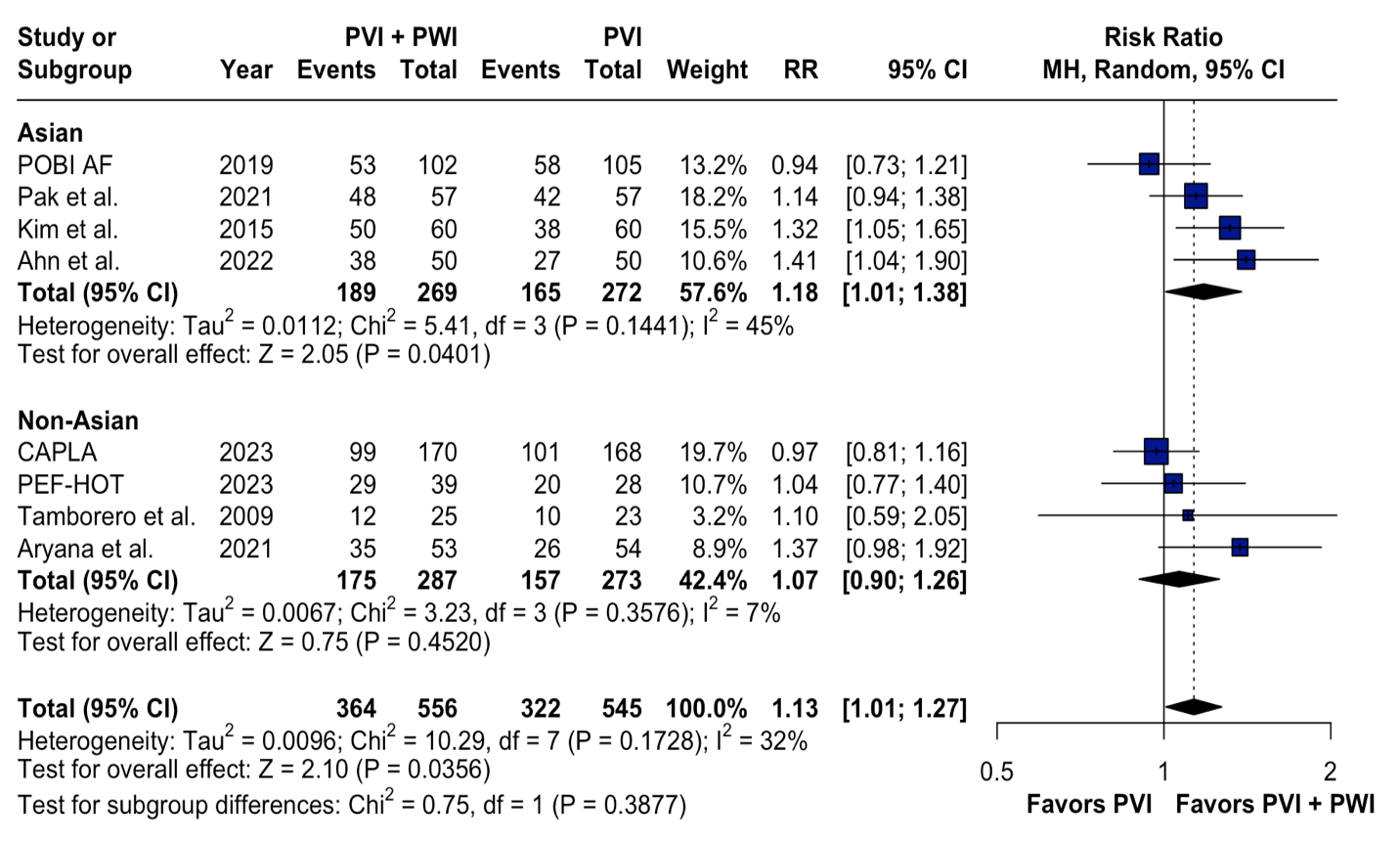


# Supplemental Figure 5. Sensitivity Analysis of the Primary Outcome

**Figure 5A.** Primary Efficacy Endpoint Omitting Studies Performing an Additional Mitral Isthmus Ablation in PWI Group.


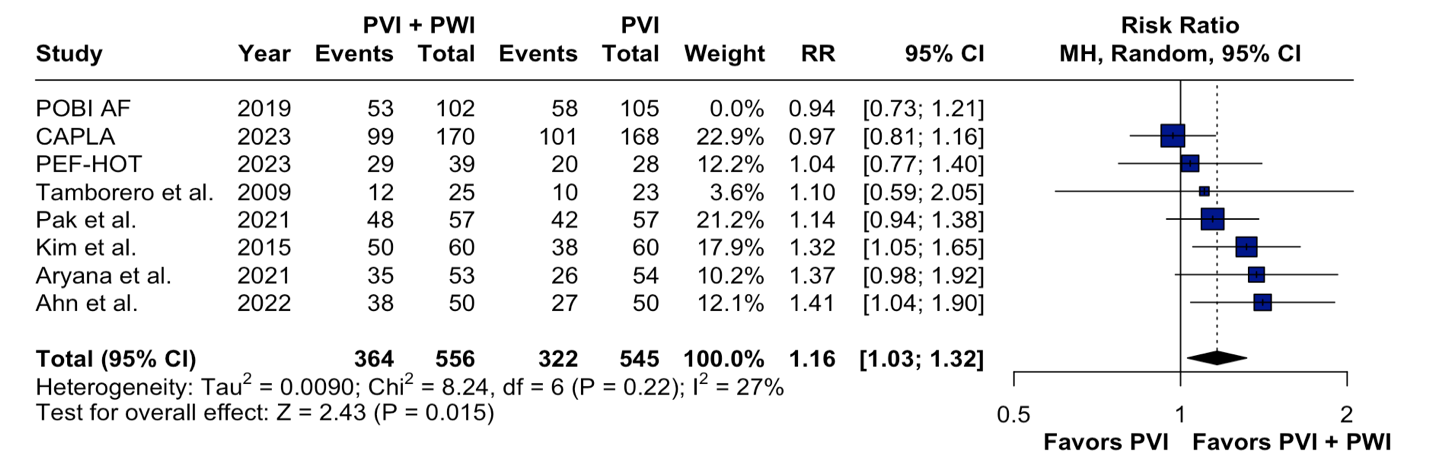


**Figure 5B.** Primary Efficacy Endpoint Under An Absolute Risk Assessment.


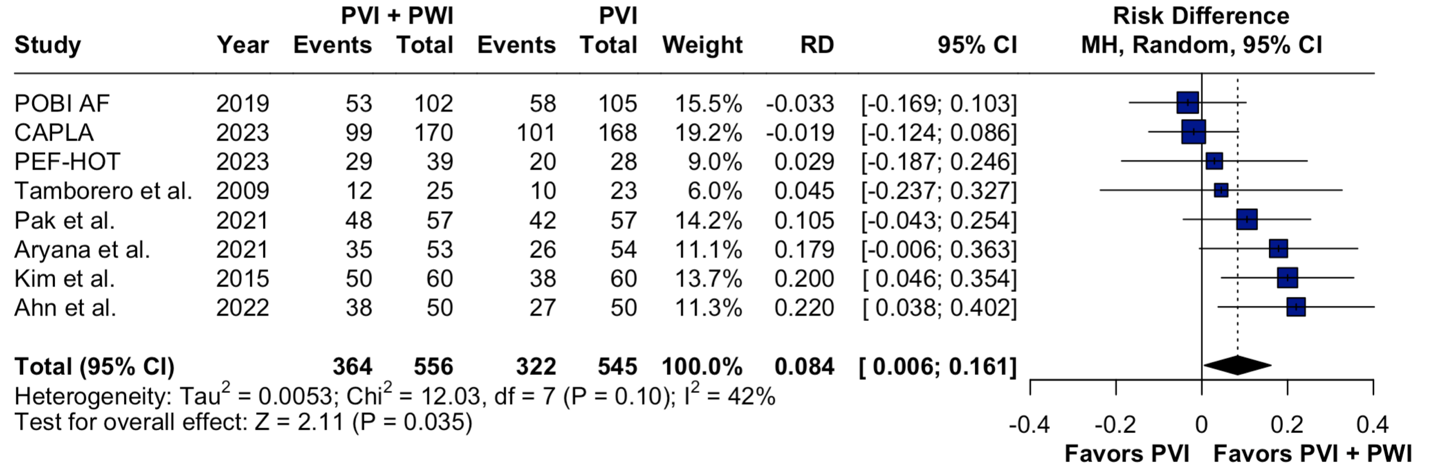


# Supplemental Figure 6. Meta-Regressions and Bubble Plots

**Figure 6A.** Meta-regression and bubble plot assessing the impact of AF duration on freedom from atrial tachyarrhythmias in all studies that reported this covariable.


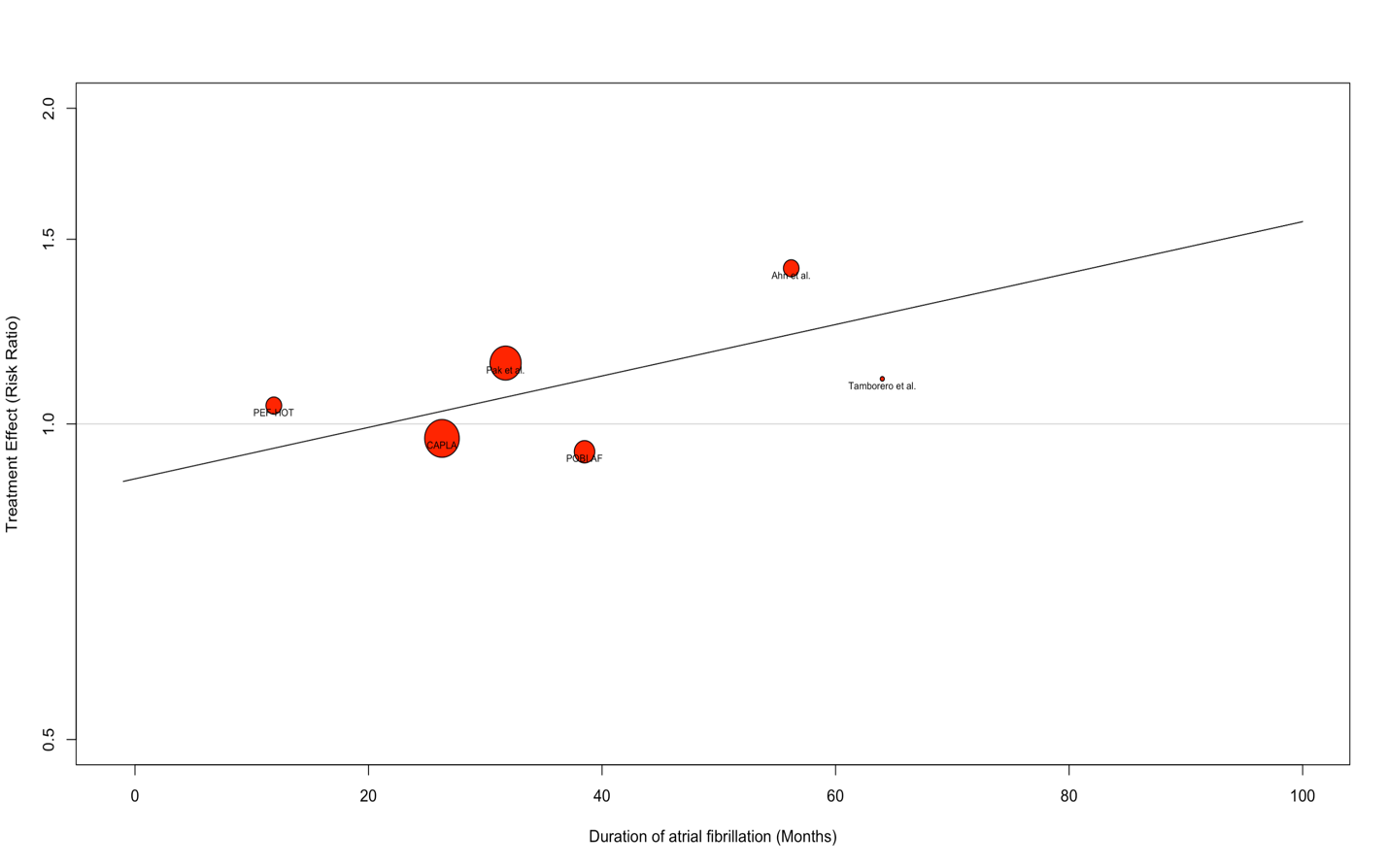


|  | Effect Estimate | p-value | I^2^ | Test for Residual Heterogeneity |
| --- | --- | --- | --- | --- |
| Intercept | -0.1206 | 0.4038 | 89% | p=0.4071 |
| AF duration | 0.0057 | 0.1732 |  |  |

**Figure 6B.** Meta-regression and bubble plot assessing the impact of mean age on freedom from atrial tachyarrhythmias in all studies that reported this covariable.


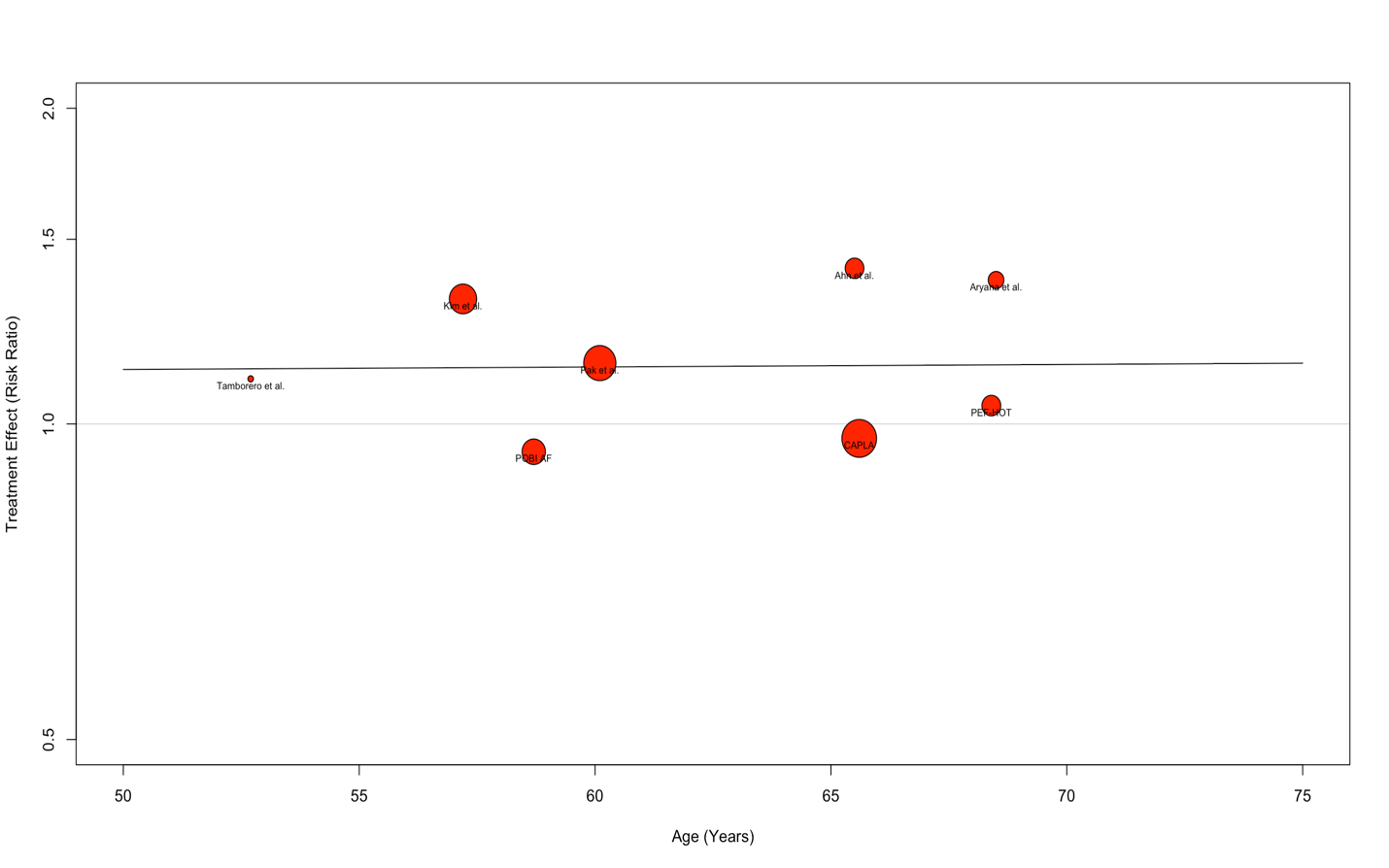


|  | Effect Estimate | p-value | I^2^ | Test for Residual Heterogeneity |
| --- | --- | --- | --- | --- |
| Intercept | 0.0912 | 0.9172 | 0% | p=0.1179 |
| Mean age | 0.0006 | 0.9677 |  |  |

**Figure 6C.** Meta-regression and bubble plot assessing the impact of left atrial diameter (LAD) on ATA recurrence in all studies that reported this covariable.


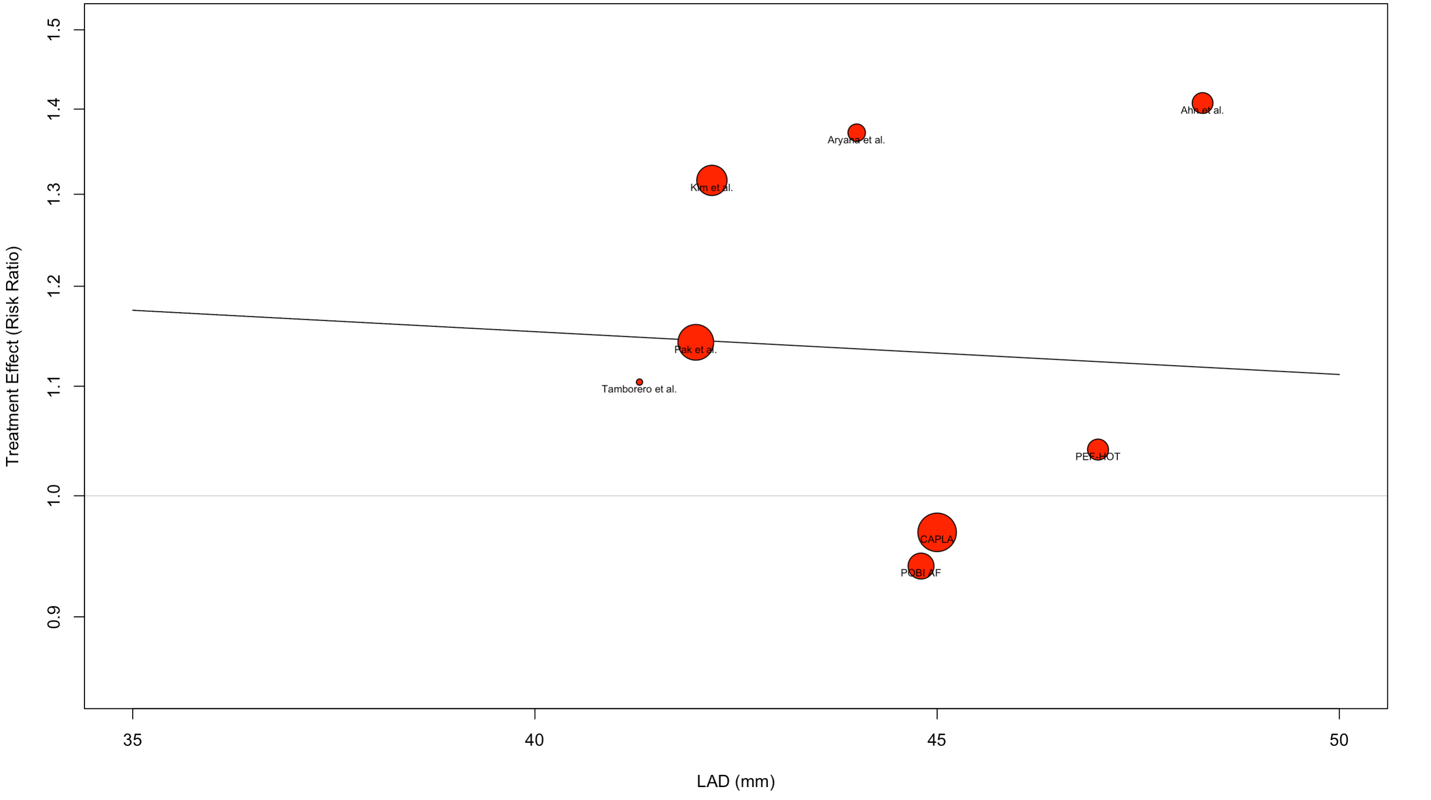


|  | Effect Estimate | p-value | I^2^ | Test for Residual Heterogeneity |
| --- | --- | --- | --- | --- |
| Intercept | 0.2922 | 0.8240 | 0% | p=0.1204 |
| LAD | -0.0037 | 0.8995 |  |  |

**Figure 6D.** Meta-regression and bubble plot assessing the impact of left ventricular ejection fraction (LVEF) on ATA recurrence in all studies that reported this covariable.


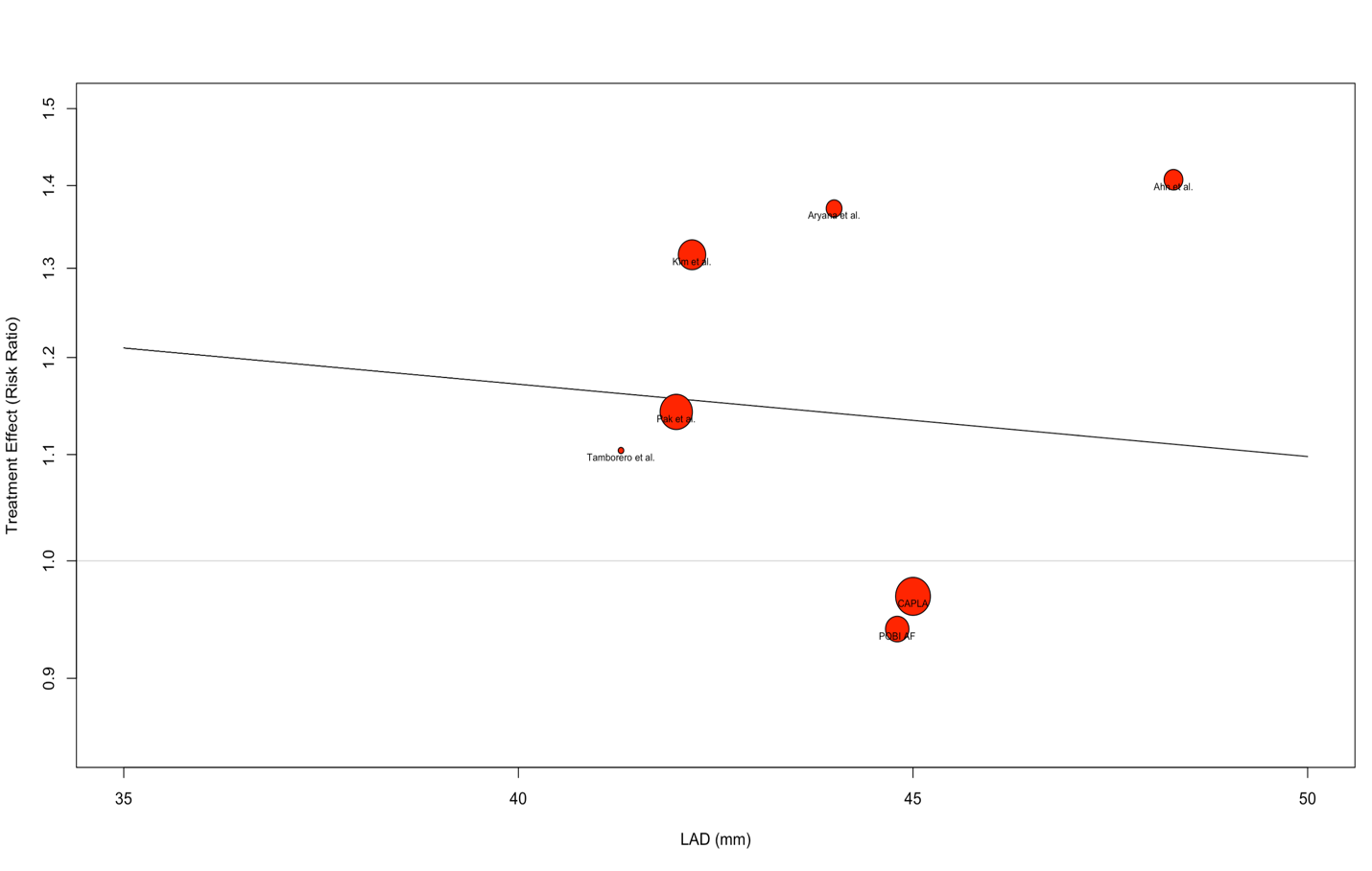


|  | Effect Estimate | p-value | I^2^ | Test for Residual Heterogeneity |
| --- | --- | --- | --- | --- |
| Intercept | -1.4117 | 0.0833 | 90.60% | p=0.3743 |
| LVEF | 0.0260 | 0.0608 |  |  |

# Supplemental Figure 7. Graphical Display of Study Heterogeneity (GOSH)

**Figure 7A.** GOSH plot.


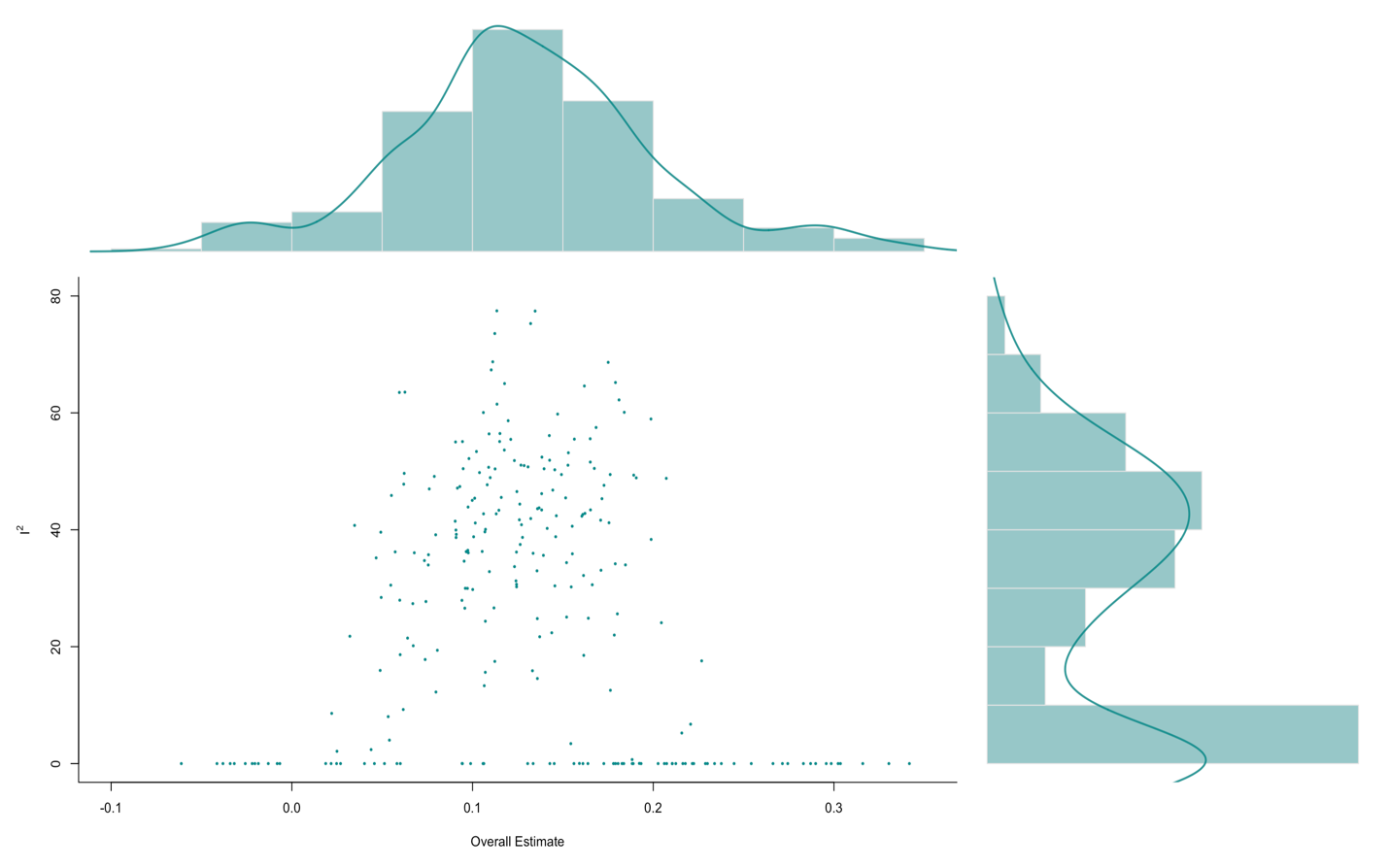


Legend: GOSH plot of I^2^ against summary effect sized (log risk ratio).

**Figure 7B.** K-means Algorithm


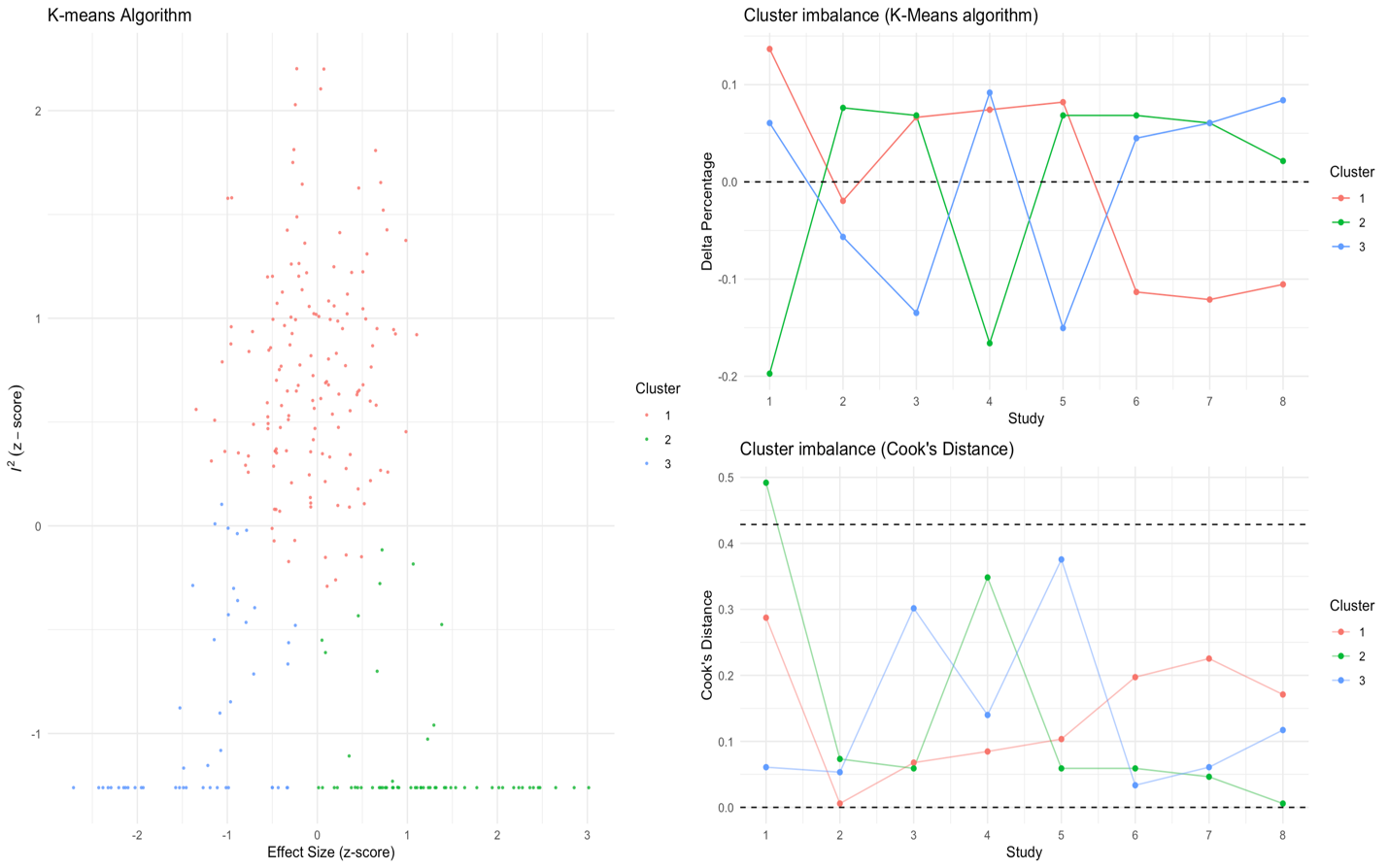


**Figure 7C.** Density-based spatial clustering of applications with noise (DBSCAN) Algorithm


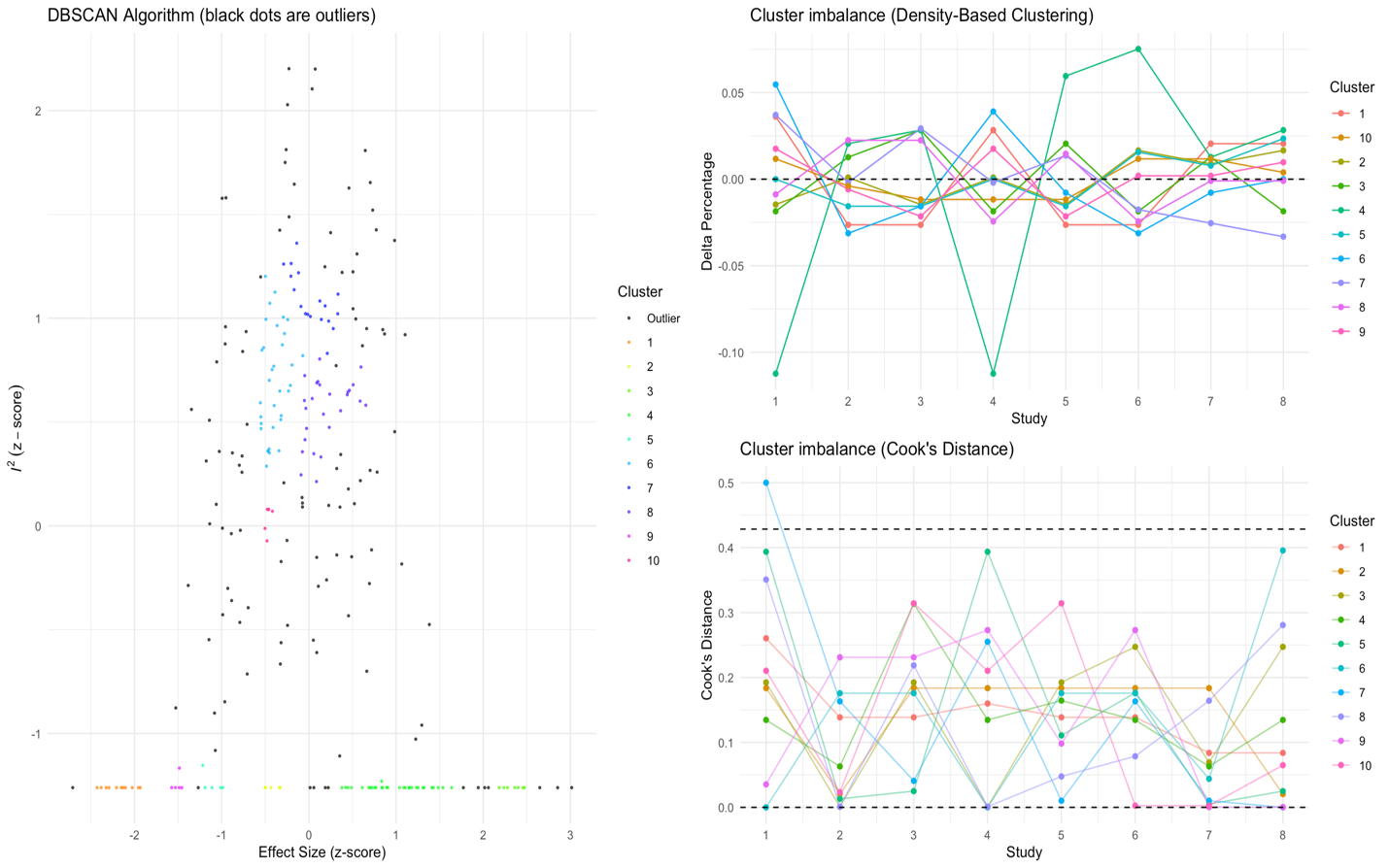


**Figure 7D.** Gaussian Mixture Model


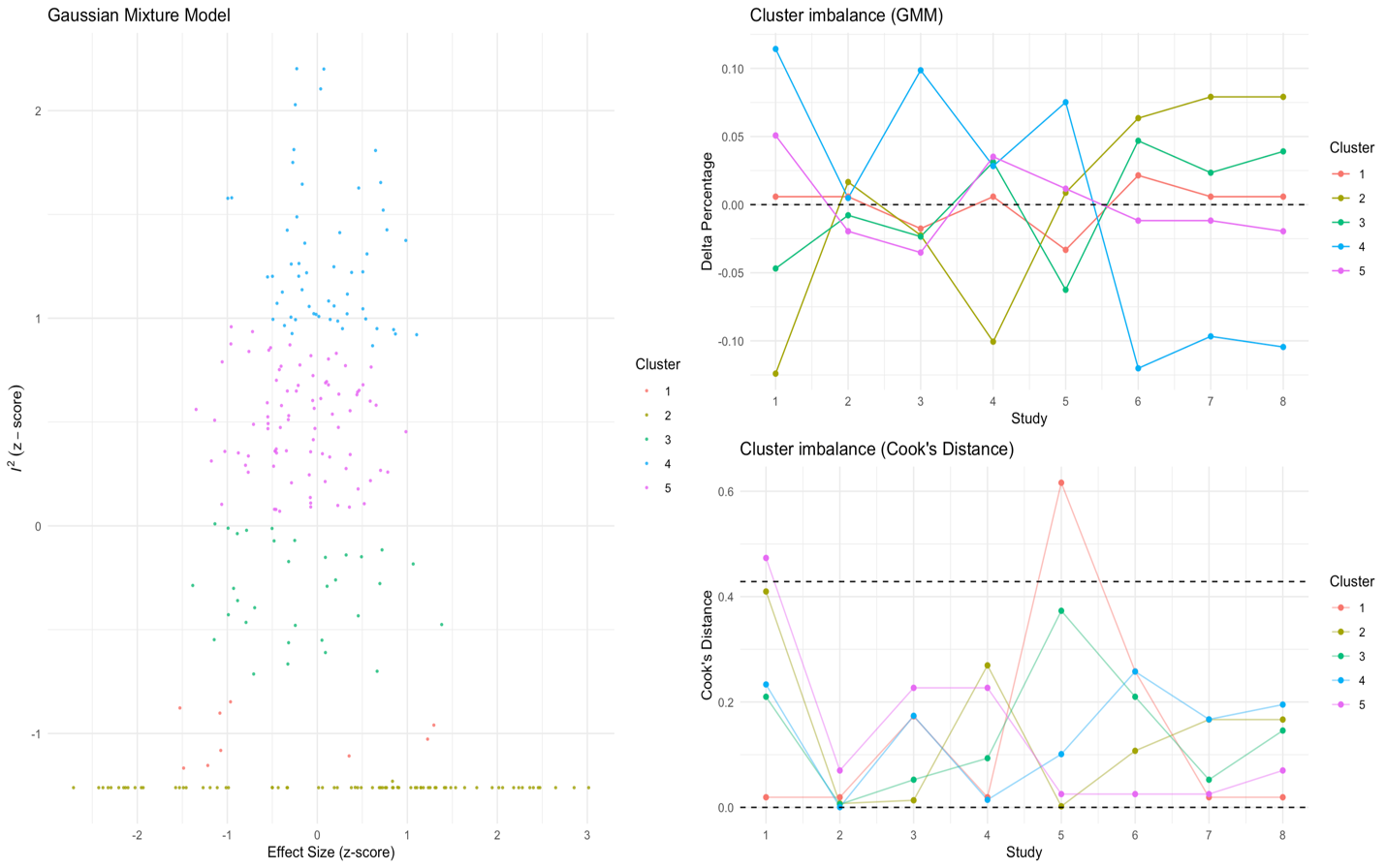


**Figure 7E**. GOSH Plot Analysis R Output for Primary Endpoint – Identification of Potential Outliers


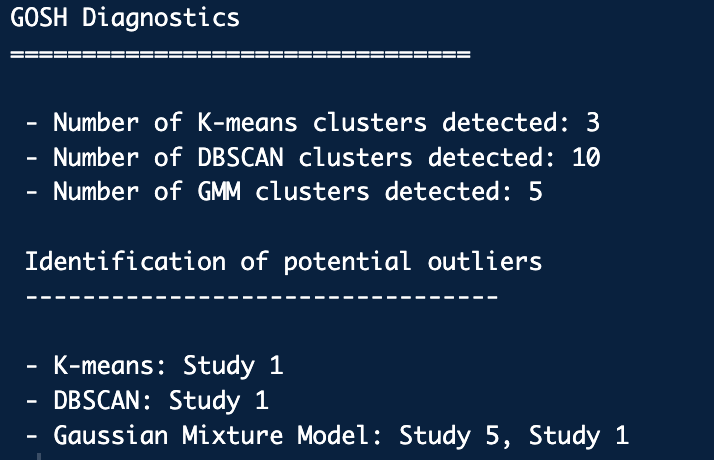


Study 1 = CAPLA 2023

**Figure 7F.** GOSH plots with the corresponding subset


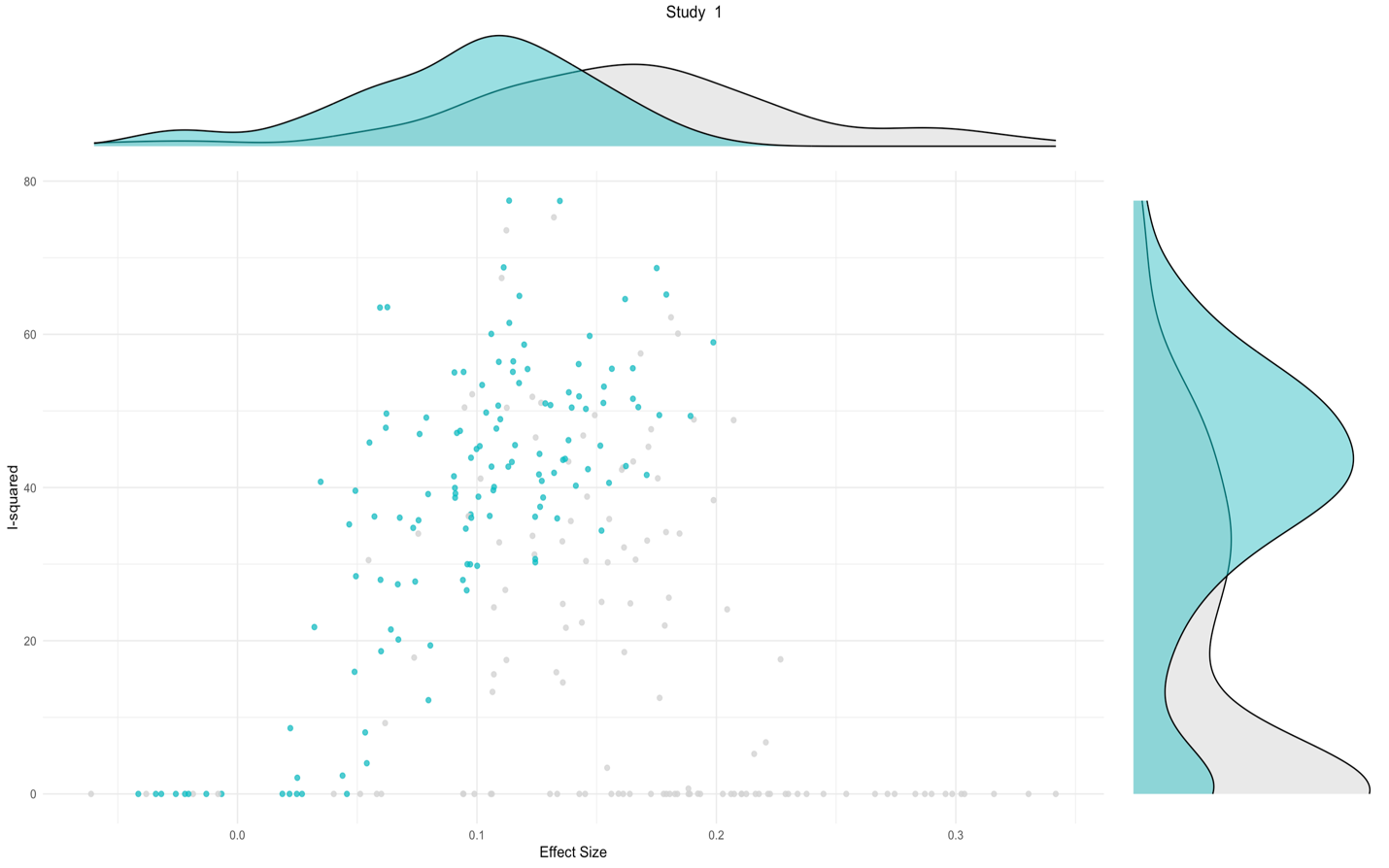


Legend: GOSH plots with the corresponding subset, including the potential outlier (CAPLA trial) colored in cyan.

# Supplemental Figure 8. Baujat Plot for the Primary Efficacy Endpoint


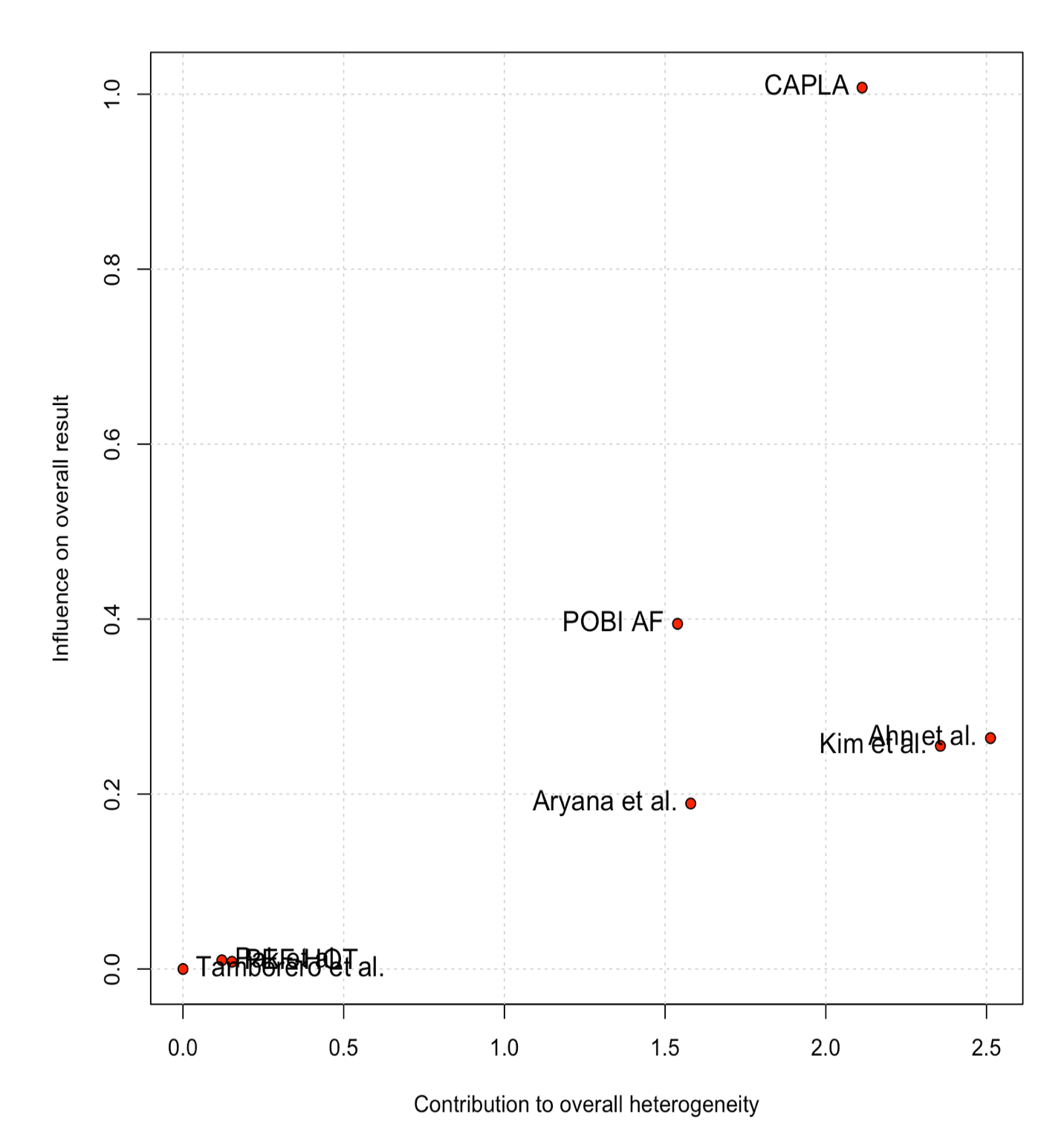


Legend: Baujat plot depicting the contribution of individual studies to overall heterogeneity on the x-axis (i.e., relative contribution to the Q-statistic) plotted against influence on the overall result on the x-axis (i.e., leave-one-out method).

# Supplemental Figure 9. Leave-one-out Analysis Sensitivity Analysis for the Primary Efficacy Endpoint


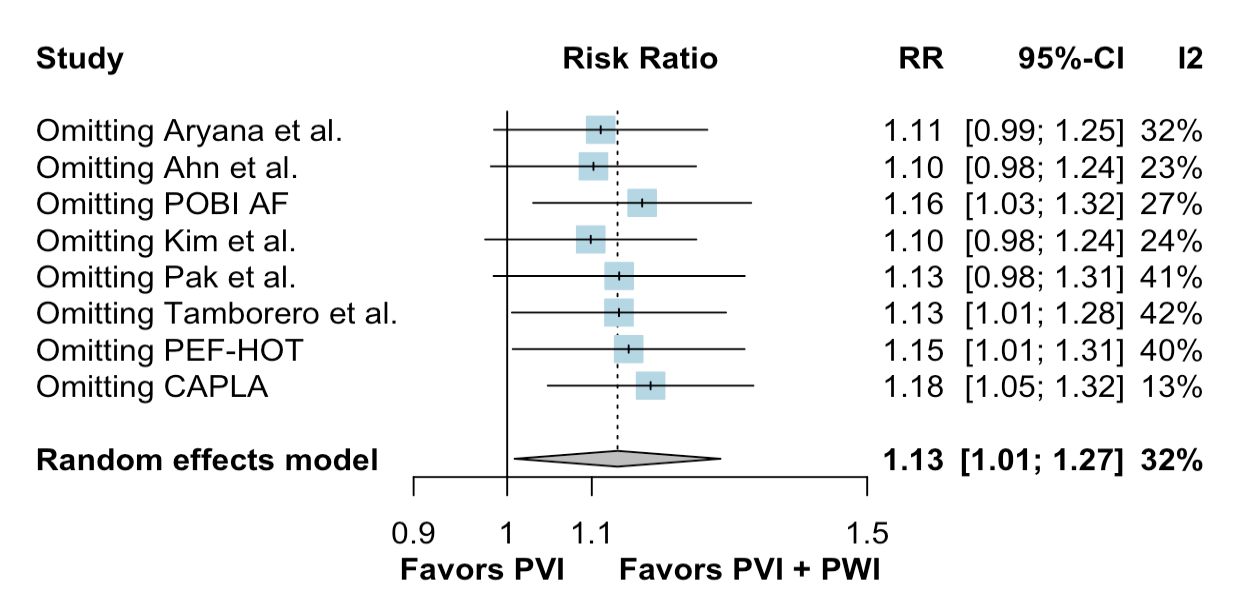


# Supplemental Figure 10. RoB 2 - Cochrane tool for assessing the risk of bias in randomized clinical trials.

**Figure 10A.** Traffic light plot


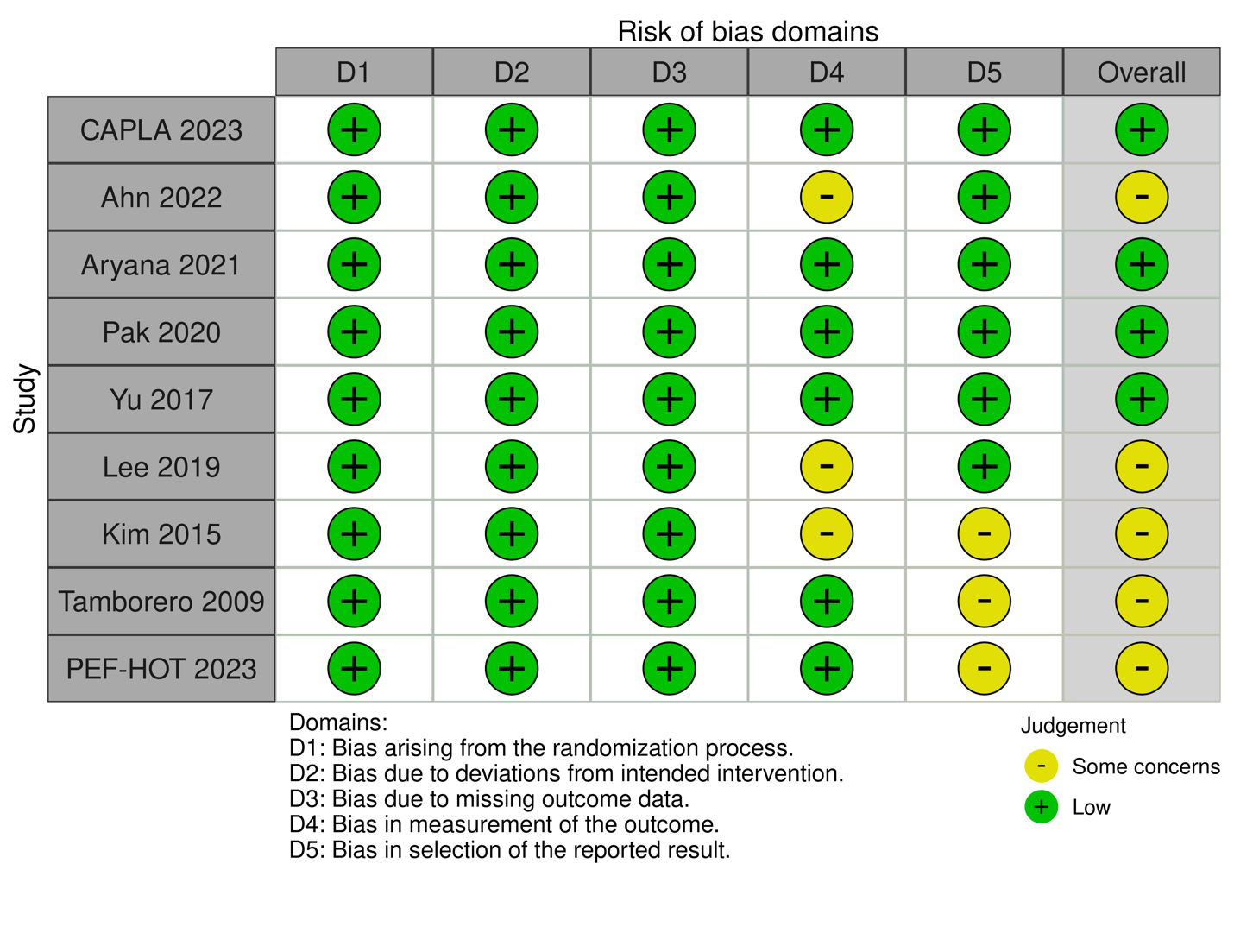


**Figure 10B.** Summary plot


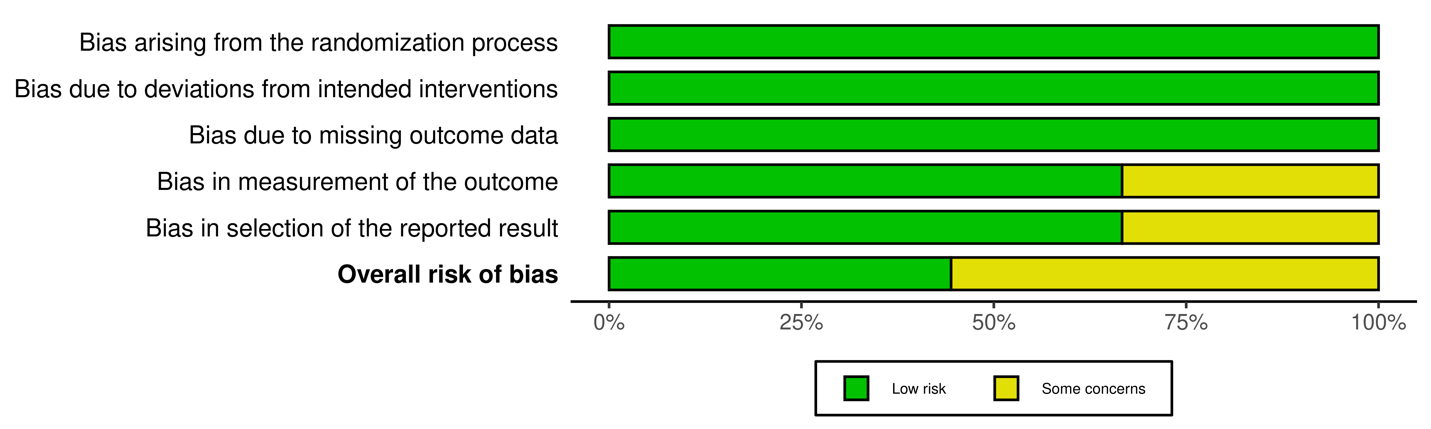


# Supplemental Figure 11. Funnel Plot and Egger’s Test for the Primary Efficacy Endpoint


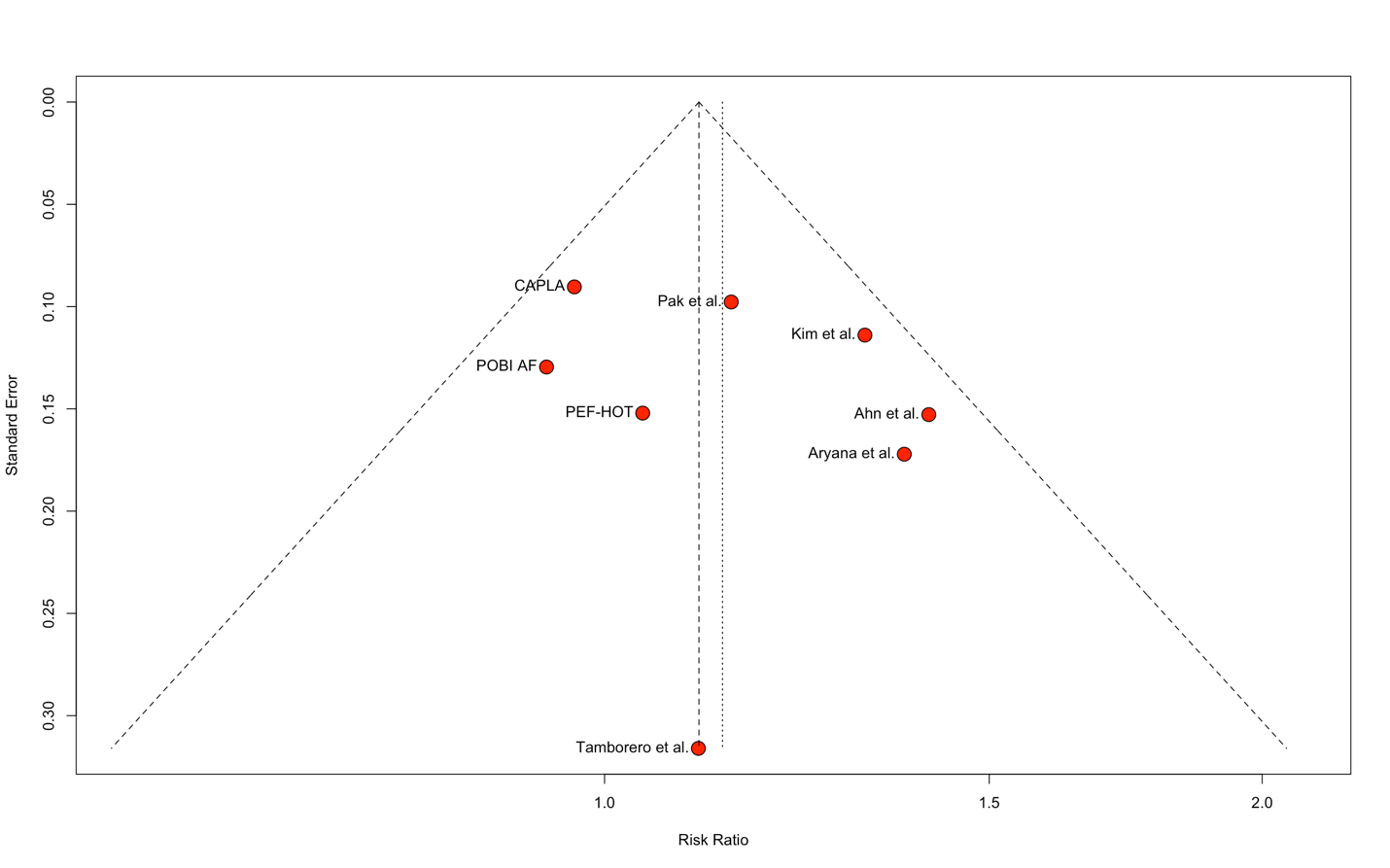


| **Egger’s Regression Test for Freedom from Atrial Tachyarrhythmia** | | | |
| --- | --- | --- | --- |
| **Intercept** | **95% CI** | **t** | **p-value** |
| 0.163 | -1.68 – 4.01 | 0.8 | 0.454 |

# Supplemental References

1. Olkin I, Dahabreh IJ, Trikalinos TA (2012) GOSH – a graphical display of study heterogeneity. Res Synth Methods 3:214–223. https://doi.org/10.1002/JRSM.1053

2. Hartigan JA, Wong MA (1979) A K-Means Clustering Algorithm. J R Stat Soc Ser C Appl Stat 28:100–108. https://doi.org/10.2307/2346830

3. Schubert E, Sander J, Ester M, et al (2017) DBSCAN Revisited, Revisited. ACM Transactions on Database Systems (TODS) 42:. https://doi.org/10.1145/3068335

4. Fraley C, Raftery AE (2002) Model-Based Clustering, Discriminant Analysis, and Density Estimation. J Am Stat Assoc 97:611–631. https://doi.org/10.1198/016214502760047131

5. Baujat B, Mahé C, Pignon JP, Hill C (2002) A graphical method for exploring heterogeneity in meta-analyses: Application to a meta-analysis of 65 trials. Stat Med 21:2641–2652. https://doi.org/10.1002/sim.1221

6. Viechtbauer W, Cheung MW-L (2010) Outlier and influence diagnostics for meta-analysis. Res Synth Methods 1:112–125. https://doi.org/10.1002/JRSM.11

7. Efthimiou O (2018) Practical guide to the meta-analysis of rare events. Evid Based Ment Health 21:72–76. https://doi.org/10.1136/EB-2018-102911

8. Sidik K, Jonkman JN (2007) A comparison of heterogeneity variance estimators in combining results of studies. Stat Med 26:1964–1981. https://doi.org/10.1002/SIM.2688

9. Sidik K, Jonkman JN (2002) A simple confidence interval for meta-analysis. Stat Med 21:3153–3159. https://doi.org/10.1002/SIM.1262

10. Knapp G, Hartung J (2003) Improved tests for a random effects meta-regression with a single covariate. Stat Med 22:2693–2710. https://doi.org/10.1002/SIM.1482

11. Higgins JPT, Thompson SG (2002) Quantifying heterogeneity in a meta-analysis. Stat Med 21:1539–1558. https://doi.org/10.1002/sim.1186

12. Mavridis D, Salanti G (2014) How to assess publication bias: funnel plot, trim-and-fill method and selection models. Evid Based Ment Health 17:30–30. https://doi.org/10.1136/EB-2013-101699

13. Viechtbauer W (2010) Conducting Meta-Analyses in R with the metafor Package. J Stat Softw 36:. https://doi.org/10.18637/jss.v036.i03

14. Harrer M, CP, FT, EDD (2019) dmetar: Companion R Package For The Guide “Doing Meta-Analysis in R”. R package version 0.0.9000. In: http://dmetar.protectlab.org/

15. Balduzzi S, Rücker G, Schwarzer G (2019) How to perform a meta-analysis with R: a practical tutorial. In: Evidence-Based Mental Health, 22nd ed. pp 153–160

16. Wickham H (2016) ggplot2: Elegant Graphics for Data Analysis. Springer New York, New York, NY
